# Supplementary material for: Global burden of disease due to rifampicin-resistant tuberculosis: a mathematical modeling analysis
Source: Nat Commun. 2023 Oct 4;14:6182. doi: 10.1038/s41467-023-41937-9 (PMC10550952; doi:10.1038/s41467-023-41937-9)
Supplement: Supplementary file 1 — Supplementary Information [file 41467_2023_41937_MOESM1_ESM.pdf]

Supplement: Global burden of disease due to rifampicin-resistant tuberculosis: a mathematical modelling analysis

Contents

p2. Table S1: Values and sources for model parameters included in uncertainty analysis.

p4. Table S2: DALYs resulting from incident RR-TB in 2020, by country.

p11. Table S3: Partial rank correlation coefficients (PRCCs) between model parameters and the total global DALYs due to RR-TB in 2020.

p12. Table S4: Global RR-TB burden of disease results for alternative analytic specifications, as compared to the main analysis.

p13. Figure S1: Data availability by country and analytic input.

p14. Supplementary methods.

p25. Supplementary citations.

Table S1: Values and sources for model parameters included in uncertainty analysis.

Parameter	Mean value	Lower bound*	Upper bound*	Prior distribution	Source
Parameters used to calculate outcomes during tuberculosis disease episode					
Total tuberculosis incidence in 2020	Country-specific values used			Gamma	[1]
Prevalence of rifampicin resistance in new tuberculosis cases	Country-specific values used			Beta	[1]
Prevalence of rifampicin resistance in previously treated tuberculosis cases	Country-specific values used			Beta	[1]
Log OR of RR-TB for males vs. females	Country-specific values used			Normal	[2]
Log OR of RR-TB for HIV infected vs. HIV uninfected	Country-specific values used			Normal	[2]
Log OR of RR-TB for age 15+ vs. age 0-14 years	Country-specific values used			Normal	[2]
Log odds of RR-TB diagnosis among individuals diagnosed with tuberculosis	Country-specific values used			Normal	[1]
Log odds of second-line treatment among individuals diagnosed with RR-TB	Country-specific values used			Normal	[1]
Disability weight for tuberculosis	0.333	0.224	0.454	Beta	[3]
Disability weight for tuberculosis and HIV	0.408	0.274	0.549	Beta	[3]
Disability weight for HIV on ART[†]	0.078	0.052	0.111	Beta	[3]
Disability weight for symptomatic HIV, no ART[†]	0.274	0.184	0.377	Beta	[3]
Duration of treated tuberculosis[‡]	1.10	0.20	2.00	Gamma	[4]
Duration of untreated tuberculosis	2.50	1.00	4.00	Gamma	[4]
Duration of treated tuberculosis, with HIV	0.51	0.01	1.00	Gamma	[4]
Duration of untreated tuberculosis, with HIV	0.11	0.01	0.2	Gamma	[4]
TB case fatality rate (overall)	Country-specific values used			Beta	[1]
Log odds ratio of tuberculosis case fatality for RR-TB on second-line regimen, vs. RS-TB on first-line regimen	Country-specific values used			Normal	[1]
Log OR of tuberculosis case fatality for HIV infected on ART, vs. HIV uninfected	0.41	0.13	0.84	Gamma	[4]
Log OR of tuberculosis case fatality for HIV infected not on ART, vs. HIV uninfected	1.25	0.73	1.9	Gamma	[4]
Log OR of tuberculosis case fatality for individuals not receiving tuberculosis treatment, vs. treated individuals	3.32	2.88	3.79	Gamma	[4]
Weight used in calculating tuberculosis case fatality for RR-TB treated with a first-line regimen	0.50	0.15	0.85	Beta	Assumed
Parameters used to calculate outcomes following survival of the tuberculosis disease episode					
Odds ratio of chronic post-TB respiratory disease for RR-TB (vs. RS-TB)[§]	1.87	1.00	2.73	Uniform	[5]
Disability weight for mild COPD	0.019	0.011	0.033	Beta	[3]
Disability weight for moderate COPD	0.225	0.153	0.31	Beta	[3]
Disability weight for severe COPD	0.408	0.273	0.556	Beta	[3]

Table S1: Values and sources for model parameters included in uncertainty analysis [continued].

Parameter	Mean value	Lower bound*	Upper bound*	Prior distribution	Source
Mortality rate ratio for post-TB individuals	2.91	2.21	3.84	Gamma	[6]
Linear term for log-linear model for mortality by FEV1%	-2.783	-3.908	-1.662	Normal	[7]
Quadratic term for log-linear model for mortality by FEV1%	0.895	0.276	1.522	Normal	[7]
Alternative mortality risk ratio for post-TB [used in sensitivity analysis]	1.78	1.61	1.98	Gamma	[8]
Intercept term for log-linear model for OR of chronic respiratory disease with post-TB	-1.494	-3.223	0.321	Normal	[9]
Slope term for log-linear model for OR of chronic respiratory disease in post-TB	0.450	0.057	0.831	Normal	[9]

* Unless otherwise specified, lower and upper bounds represent the 2.5 and 97.5 percentiles of the parameter distribution. Together these represent an equal-tailed 95% interval for the parameter. § Ranges represent upper and lower bounds of uniform distribution. † Disability weights for HIV without tuberculosis are applied to HIV-infected tuberculosis survivors. ‡ Duration values assumed to represent the total duration of disability during tuberculosis disease episode. OR = odds ratio. RR-TB = rifampicin-resistant tuberculosis. RS-TB = rifampicin-susceptible tuberculosis. ART = antiretroviral therapy for HIV. FEV1% = Forced expiratory volume over 1 second compared to predicted value. COPD = chronic obstructive pulmonary disease.

Table S2: DALYs resulting from incident RR-TB in 2020, by country.

Country	ISO3 code	Rank	RR-TB DALYs per 100,000	DALYs per RR-TB case	Total RR-TB DALYs (thousands)	Percent of global RR-TB burden (%)
India	IND	1	119.4 (91.2, 147.5)	20.1 (15.8, 24.2)	1666.75 (1273.53, 2059.59)	24.095 (20.297, 28.195)
Russian Federation	RUS	2	316.3 (240.7, 413.6)	14.7 (11.0, 18.1)	460.56 (350.51, 602.33)	6.661 (5.434, 8.307)
Indonesia	IDN	3	167.6 (113.2, 241.9)	18.9 (15.0, 22.5)	455.52 (307.86, 657.70)	6.574 (4.663, 8.812)
Philippines	PHL	4	392.9 (220.1, 658.8)	16.5 (11.4, 22.0)	440.76 (246.94, 739.12)	6.318 (3.956, 9.827)
Pakistan	PAK	5	190.4 (80.5, 358.4)	13.4 (9.2, 17.4)	432.66 (183.00, 814.19)	6.182 (2.911, 10.738)
South Africa	ZAF	6	702.9 (448.6, 1030.4)	21.0 (13.9, 28.7)	413.30 (263.81, 605.87)	5.966 (4.080, 8.301)
Nigeria	NGA	7	160.5 (41.6, 391.3)	24.4 (17.0, 32.9)	334.31 (86.71, 815.10)	4.776 (1.286, 10.947)
China	CHN	8	22.9 (15.2, 31.7)	8.7 (5.7, 11.7)	326.97 (216.94, 452.26)	4.698 (3.574, 5.976)
Myanmar	MMR	9	383.0 (259.2, 531.0)	21.2 (14.7, 28.9)	204.61 (138.46, 283.68)	2.961 (2.131, 4.045)
Ukraine	UKR	10	383.3 (249.2, 577.0)	16.1 (12.0, 20.8)	168.32 (109.40, 253.36)	2.435 (1.636, 3.561)
Democratic Republic of the Congo	COD	11	152.0 (79.6, 252.3)	24.2 (15.3, 35.9)	141.15 (73.95, 234.30)	2.042 (1.112, 3.370)
Mozambique	MOZ	12	416.8 (271.0, 583.5)	26.0 (15.8, 35.0)	129.95 (84.51, 181.93)	1.885 (1.237, 2.648)
Angola	AGO	13	327.7 (36.9, 1096.4)	22.9 (15.4, 32.9)	109.54 (12.35, 366.52)	1.563 (0.184, 4.793)
Viet Nam	VNM	14	104.2 (66.0, 157.7)	11.9 (8.1, 15.8)	100.72 (63.76, 152.46)	1.448 (1.020, 2.030)
Afghanistan	AFG	15	235.2 (23.9, 770.9)	21.9 (13.7, 34.9)	91.66 (9.32, 300.42)	1.322 (0.139, 4.450)
Democratic People's Republic of Korea	PRK	16	352.2 (152.6, 673.3)	20.3 (15.1, 25.6)	91.09 (39.48, 174.15)	1.317 (0.575, 2.439)
Bangladesh	BGD	17	39.8 (21.9, 67.4)	15.3 (10.4, 21.7)	66.65 (36.59, 112.91)	0.961 (0.560, 1.536)
Kazakhstan	KAZ	18	324.2 (225.3, 461.7)	11.8 (8.2, 15.3)	61.54 (42.76, 87.62)	0.887 (0.674, 1.170)
Ethiopia	ETH	19	51.5 (33.9, 71.9)	24.8 (15.7, 34.8)	60.35 (39.71, 84.21)	0.875 (0.600, 1.233)
United Republic of Tanzania	TZA	20	80.4 (44.6, 132.2)	25.2 (14.3, 37.9)	49.64 (27.54, 81.59)	0.720 (0.414, 1.187)
Papua New Guinea	PNG	21	507.6 (222.0, 921.5)	25.7 (18.1, 34.5)	49.49 (21.64, 89.84)	0.715 (0.329, 1.228)
Somalia	SOM	22	289.2 (89.1, 663.0)	24.2 (15.6, 35.2)	47.83 (14.74, 109.64)	0.692 (0.216, 1.545)
Nepal	NPL	23	162.2 (80.5, 286.2)	17.6 (10.7, 26.8)	47.62 (23.64, 84.01)	0.689 (0.365, 1.152)
Uzbekistan	UZB	24	133.8 (84.0, 200.5)	10.6 (7.2, 14.0)	44.86 (28.18, 67.21)	0.645 (0.443, 0.922)
Zambia	ZMB	25	224.2 (147.6, 336.1)	22.0 (15.3, 30.0)	42.44 (27.95, 63.63)	0.613 (0.423, 0.878)
Kenya	KEN	26	75.5 (44.5, 115.7)	26.1 (17.9, 35.5)	39.24 (23.14, 60.16)	0.569 (0.350, 0.887)
Côte d'Ivoire	CIV	27	145.6 (78.7, 251.2)	22.5 (14.9, 31.5)	39.03 (21.10, 67.36)	0.565 (0.298, 0.966)

Table S2: DALYs resulting from incident RR-TB in 2020, by country [continued]

Country	ISO3 code	Rank	RR-TB DALYs per 100,000	DALYs per RR-TB case	Total RR-TB DALYs (thousands)	Percent of global RR-TB burden (%)
Brazil	BRA	28	17.4 (5.4, 37.1)	15.0 (10.4, 19.9)	37.02 (11.48, 79.13)	0.534 (0.167, 1.098)
Thailand	THA	29	50.0 (33.3, 72.3)	14.5 (10.0, 19.4)	35.73 (23.78, 51.68)	0.516 (0.363, 0.714)
Tajikistan	TJK	30	371.5 (262.4, 503.5)	15.0 (11.2, 18.9)	35.45 (25.04, 48.05)	0.512 (0.387, 0.673)
Uganda	UGA	31	77.7 (31.9, 168.9)	21.2 (14.1, 31.0)	34.50 (14.16, 74.99)	0.496 (0.213, 1.016)
Ghana	GHA	32	92.6 (38.6, 177.4)	27.1 (14.8, 41.7)	29.79 (12.43, 57.09)	0.433 (0.175, 0.892)
Peru	PER	33	81.0 (54.8, 112.0)	12.7 (9.2, 16.3)	26.97 (18.26, 37.32)	0.389 (0.288, 0.507)
Kyrgyzstan	KGZ	34	413.4 (285.7, 553.5)	11.7 (8.2, 15.1)	26.56 (18.36, 35.56)	0.382 (0.304, 0.464)
Turkmenistan	TKM	35	397.3 (280.8, 535.5)	19.9 (15.1, 24.8)	24.83 (17.55, 33.47)	0.360 (0.258, 0.488)
Cameroon	CMR	36	80.7 (39.2, 145.1)	25.1 (16.8, 35.9)	21.39 (10.39, 38.44)	0.310 (0.156, 0.549)
Malawi	MWI	37	106.3 (54.3, 193.8)	29.2 (16.4, 42.4)	20.60 (10.53, 37.55)	0.299 (0.149, 0.553)
Zimbabwe	ZWE	38	127.1 (89.3, 179.9)	24.7 (17.7, 31.8)	19.91 (14.00, 28.18)	0.289 (0.205, 0.406)
Madagascar	MDG	39	65.4 (15.9, 177.7)	22.9 (14.8, 34.7)	18.46 (4.48, 50.15)	0.267 (0.065, 0.726)
Lesotho	LSO	40	773.0 (444.0, 1225.1)	27.5 (19.2, 36.3)	17.42 (10.01, 27.62)	0.253 (0.148, 0.405)
Gabon	GAB	41	693.9 (201.8, 2368.0)	27.7 (17.6, 39.5)	15.91 (4.63, 54.29)	0.231 (0.066, 0.811)
Haiti	HTI	42	139.1 (20.1, 383.7)	17.5 (12.4, 22.7)	15.73 (2.27, 43.38)	0.227 (0.034, 0.619)
Cambodia	KHM	43	94.6 (49.7, 162.6)	15.9 (10.7, 22.0)	15.51 (8.14, 26.66)	0.224 (0.122, 0.376)
Sudan	SDN	44	34.6 (16.6, 62.2)	16.5 (9.9, 25.9)	15.38 (7.37, 27.63)	0.222 (0.111, 0.397)
Sierra Leone	SLE	45	182.0 (12.8, 614.5)	24.0 (15.9, 34.1)	14.99 (1.05, 50.60)	0.216 (0.015, 0.724)
Mongolia	MNG	46	437.1 (193.2, 782.8)	13.9 (8.7, 19.0)	14.40 (6.37, 25.79)	0.207 (0.101, 0.355)
Morocco	MAR	47	39.2 (20.3, 66.7)	18.3 (12.2, 26.0)	14.38 (7.44, 24.49)	0.208 (0.112, 0.357)
Central African Republic	CAF	48	267.1 (34.5, 754.0)	25.5 (17.8, 34.4)	14.27 (1.85, 40.29)	0.207 (0.027, 0.608)
Republic of Moldova	MDA	49	456.4 (321.9, 599.5)	13.1 (9.6, 16.0)	14.08 (9.93, 18.50)	0.203 (0.162, 0.252)
Congo	COG	50	243.7 (28.6, 799.2)	27.8 (18.6, 39.0)	13.90 (1.63, 45.57)	0.202 (0.025, 0.674)
Belarus	BLR	51	143.8 (96.9, 199.9)	12.3 (9.0, 15.4)	13.85 (9.33, 19.26)	0.200 (0.143, 0.275)
Liberia	LBR	52	270.0 (6.1, 1173.5)	25.5 (16.6, 36.4)	13.74 (0.31, 59.70)	0.198 (0.004, 0.815)
Guinea	GIN	53	99.8 (23.9, 286.0)	19.4 (13.2, 28.4)	13.18 (3.15, 37.76)	0.190 (0.050, 0.543)
Namibia	NAM	54	527.7 (364.7, 726.9)	23.0 (16.2, 30.0)	13.13 (9.08, 18.09)	0.190 (0.135, 0.268)
Mexico	MEX	55	10.4 (4.3, 21.1)	13.7 (9.6, 18.2)	13.06 (5.42, 26.63)	0.189 (0.078, 0.378)

Table S2: DALYs resulting from incident RR-TB in 2020, by country [continued]

Country	ISO3 code	Rank	RR-TB DALYs per 100,000	DALYs per RR-TB case	Total RR-TB DALYs (thousands)	Percent of global RR-TB burden (%)
Niger	NER	56	53.5<br>(26.5, 95.0)	20.6<br>(13.2, 31.2)	13.01<br>(6.46, 23.11)	0.188<br>(0.097, 0.330)
Algeria	DZA	57	29.9<br>(4.7, 73.3)	14.6<br>(9.3, 21.4)	13.00<br>(2.06, 31.85)	0.188<br>(0.032, 0.450)
Azerbaijan	AZE	58	124.2<br>(89.0, 167.4)	14.3<br>(10.8, 17.9)	12.77<br>(9.15, 17.22)	0.185<br>(0.141, 0.239)
Colombia	COL	59	23.3<br>(4.9, 54.2)	12.4<br>(8.6, 16.6)	11.86<br>(2.49, 27.62)	0.171<br>(0.035, 0.406)
South Sudan	SSD	60	108.4<br>(5.0, 376.5)	22.8<br>(15.2, 34.9)	11.49<br>(0.53, 39.93)	0.167<br>(0.008, 0.562)
Chad	TCD	61	56.8<br>(34.8, 85.6)	25.5<br>(16.6, 36.0)	9.45<br>(5.80, 14.25)	0.137<br>(0.087, 0.209)
Republic of Korea	KOR	62	17.0<br>(12.0, 21.7)	8.9<br>(6.4, 11.3)	8.80<br>(6.24, 11.26)	0.127<br>(0.100, 0.155)
Iraq	IRQ	63	20.6<br>(11.6, 34.8)	11.0<br>(7.6, 14.4)	8.75<br>(4.93, 14.82)	0.126<br>(0.074, 0.198)
Botswana	BWA	64	338.9<br>(117.3, 701.2)	26.7<br>(20.0, 34.3)	8.63<br>(2.99, 17.86)	0.125<br>(0.042, 0.266)
Yemen	YEM	65	26.3<br>(9.8, 52.8)	20.6<br>(14.3, 27.3)	8.50<br>(3.16, 17.04)	0.123<br>(0.045, 0.246)
Romania	ROU	66	42.4<br>(32.8, 53.5)	15.5<br>(12.2, 18.8)	8.25<br>(6.37, 10.41)	0.120<br>(0.094, 0.149)
Malaysia	MYS	67	23.9<br>(16.2, 32.7)	14.2<br>(9.7, 19.1)	7.94<br>(5.38, 10.87)	0.115<br>(0.086, 0.150)
Venezuela (Bolivarian Republic of)	VEN	68	27.8<br>(3.5, 79.7)	14.6<br>(9.9, 20.7)	7.93<br>(1.00, 22.71)	0.115<br>(0.014, 0.324)
Eswatini	SWZ	69	577.9<br>(333.7, 911.5)	23.9<br>(15.4, 33.2)	6.82<br>(3.94, 10.76)	0.099<br>(0.060, 0.153)
Burundi	BDI	70	55.8<br>(16.8, 162.3)	23.0<br>(14.6, 33.4)	6.82<br>(2.05, 19.84)	0.099<br>(0.030, 0.289)
Japan	JPN	71	4.4<br>(0.7, 11.6)	10.5<br>(8.0, 12.9)	5.51<br>(0.91, 14.50)	0.080<br>(0.013, 0.215)
Ecuador	ECU	72	30.6<br>(20.5, 43.5)	13.4<br>(9.5, 17.5)	5.38<br>(3.61, 7.65)	0.078<br>(0.055, 0.110)
Equatorial Guinea	GNQ	73	333.3<br>(99.6, 1036.2)	30.1<br>(20.5, 41.0)	5.32<br>(1.59, 16.54)	0.077<br>(0.024, 0.237)
Senegal	SEN	74	30.1<br>(11.4, 62.8)	19.2<br>(12.3, 28.3)	4.95<br>(1.87, 10.33)	0.072<br>(0.028, 0.145)
Dominican Republic	DOM	75	44.0<br>(26.1, 63.5)	14.0<br>(8.7, 19.6)	4.84<br>(2.87, 6.98)	0.070<br>(0.046, 0.099)
Guinea-Bissau	GNB	76	232.2<br>(127.8, 366.2)	30.4<br>(20.7, 41.2)	4.68<br>(2.58, 7.38)	0.068<br>(0.038, 0.107)
Bolivia (Plurinational State of)	BOL	77	37.1<br>(22.6, 56.7)	15.7<br>(11.0, 21.7)	4.43<br>(2.70, 6.76)	0.064<br>(0.039, 0.099)
Georgia	GEO	78	107.1<br>(70.3, 147.5)	10.0<br>(6.8, 13.2)	4.03<br>(2.65, 5.56)	0.058<br>(0.042, 0.074)
Argentina	ARG	79	8.1<br>(1.5, 22.2)	11.1<br>(7.5, 14.7)	3.65<br>(0.66, 10.01)	0.053<br>(0.009, 0.140)
Bhutan	BTN	80	455.2<br>(303.0, 643.4)	20.7<br>(14.1, 28.0)	3.52<br>(2.34, 4.97)	0.051<br>(0.036, 0.070)
Türkiye	TUR	81	3.8<br>(2.4, 5.4)	8.6<br>(5.5, 11.8)	3.23<br>(1.99, 4.55)	0.047<br>(0.031, 0.062)
Mali	MLI	82	14.9<br>(8.4, 25.1)	21.1<br>(13.4, 31.0)	3.16<br>(1.79, 5.32)	0.046<br>(0.026, 0.078)
Burkina Faso	BFA	83	14.4<br>(8.3, 23.3)	17.9<br>(11.5, 25.7)	3.09<br>(1.78, 5.01)	0.045<br>(0.026, 0.072)

Table S2: DALYs resulting from incident RR-TB in 2020, by country [continued]

Country	ISO3 code	Rank	RR-TB DALYs per 100,000	DALYs per RR-TB case	Total RR-TB DALYs (thousands)	Percent of global RR-TB burden (%)
Lao People's Democratic Republic	LAO	84	36.9 (24.1, 53.8)	21.4 (13.1, 30.6)	2.70 (1.76, 3.94)	0.039 (0.026, 0.059)
Gambia	GMB	85	99.8 (14.8, 299.6)	25.8 (18.0, 35.7)	2.57 (0.38, 7.71)	0.037 (0.005, 0.113)
Rwanda	RWA	86	19.1 (12.9, 26.5)	19.5 (13.4, 26.5)	2.51 (1.69, 3.48)	0.036 (0.026, 0.050)
Saudi Arabia	SAU	87	7.0 (2.3, 14.5)	34.9 (25.4, 42.5)	2.51 (0.82, 5.22)	0.037 (0.012, 0.078)
Lithuania	LTU	88	83.2 (64.7, 101.2)	14.8 (11.4, 18.0)	2.35 (1.82, 2.85)	0.034 (0.027, 0.042)
Iran (Islamic Republic of)	IRN	89	2.5 (1.2, 4.7)	10.4 (7.0, 14.6)	2.22 (1.04, 4.08)	0.032 (0.016, 0.056)
Mauritania	MRT	90	46.3 (14.9, 133.3)	18.9 (12.0, 27.8)	2.08 (0.67, 6.00)	0.030 (0.010, 0.087)
Eritrea	ERI	91	56.7 (26.0, 105.7)	21.6 (10.8, 36.0)	2.02 (0.93, 3.76)	0.029 (0.014, 0.055)
Benin	BEN	92	14.9 (8.2, 23.4)	23.3 (14.1, 35.0)	1.88 (1.03, 2.96)	0.027 (0.015, 0.044)
Egypt	EGY	93	1.7 (0.9, 2.6)	7.5 (4.2, 11.0)	1.86 (1.02, 2.81)	0.027 (0.016, 0.039)
Guatemala	GTM	94	10.4 (6.8, 14.8)	15.0 (10.5, 19.8)	1.81 (1.18, 2.57)	0.026 (0.018, 0.037)
Djibouti	DJI	95	132.9 (53.4, 268.4)	18.3 (12.4, 26.1)	1.45 (0.58, 2.93)	0.021 (0.008, 0.040)
Armenia	ARM	96	50.7 (30.9, 74.5)	9.8 (6.5, 13.0)	1.42 (0.87, 2.09)	0.020 (0.014, 0.029)
Spain	ESP	97	2.9 (1.2, 5.4)	9.3 (5.9, 13.4)	1.35 (0.55, 2.58)	0.019 (0.009, 0.036)
Germany	DEU	98	1.6 (0.9, 2.3)	10.2 (6.3, 14.0)	1.30 (0.77, 1.92)	0.019 (0.012, 0.027)
United Kingdom	GBR	99	1.9 (1.2, 2.7)	13.7 (8.8, 19.3)	1.28 (0.80, 1.84)	0.019 (0.012, 0.027)
Libya	LBY	100	18.9 (7.8, 37.0)	19.0 (11.6, 28.9)	1.26 (0.52, 2.46)	0.018 (0.008, 0.037)
Panama	PAN	101	28.5 (16.3, 44.9)	18.3 (12.1, 25.3)	1.22 (0.70, 1.93)	0.018 (0.010, 0.028)
Timor-Leste	TLS	102	89.6 (34.6, 173.9)	20.9 (14.1, 28.8)	1.16 (0.45, 2.26)	0.017 (0.006, 0.032)
United States of America	USA	103	0.3 (0.2, 0.5)	8.1 (4.8, 12.2)	1.09 (0.63, 1.61)	0.016 (0.010, 0.022)
Honduras	HND	104	10.7 (2.3, 27.5)	18.2 (12.7, 25.4)	1.08 (0.23, 2.78)	0.016 (0.003, 0.041)
Chile	CHL	105	5.3 (3.7, 7.4)	14.0 (9.7, 18.9)	1.02 (0.71, 1.42)	0.015 (0.010, 0.021)
Italy	ITA	106	1.7 (1.1, 2.5)	9.0 (6.3, 12.1)	1.00 (0.63, 1.49)	0.014 (0.010, 0.021)
Latvia	LVA	107	41.4 (31.8, 52.3)	17.6 (14.0, 21.5)	0.79 (0.60, 0.99)	0.011 (0.009, 0.014)
Sri Lanka	LKA	108	3.6 (1.1, 8.5)	8.9 (6.0, 11.8)	0.78 (0.24, 1.86)	0.011 (0.004, 0.028)
El Salvador	SLV	109	10.0 (2.8, 22.5)	10.0 (6.3, 13.7)	0.63 (0.18, 1.42)	0.009 (0.003, 0.020)
Cuba	CUB	110	5.1 (2.5, 9.0)	14.5 (9.4, 20.5)	0.57 (0.28, 1.02)	0.008 (0.004, 0.015)
Nicaragua	NIC	111	8.4 (5.0, 12.9)	12.2 (8.2, 16.6)	0.57 (0.34, 0.87)	0.008 (0.005, 0.013)

Table S2: DALYs resulting from incident RR-TB in 2020, by country [continued]

Country	ISO3 code	Rank	RR-TB DALYs per 100,000	DALYs per RR-TB case	Total RR-TB DALYs (thousands)	Percent of global RR-TB burden (%)
Poland	POL	112	1.5 (1.0, 2.1)	13.7 (9.5, 18.5)	0.56 (0.38, 0.79)	0.008 (0.006, 0.011)
Tunisia	TUN	113	4.3 (2.1, 7.5)	8.0 (5.1, 11.1)	0.52 (0.25, 0.91)	0.007 (0.004, 0.012)
Togo	TGO	114	5.9 (3.7, 8.6)	15.9 (10.4, 22.7)	0.50 (0.31, 0.73)	0.007 (0.005, 0.010)
Syrian Arab Republic	SYR	115	2.3 (1.1, 3.9)	7.7 (4.0, 11.8)	0.49 (0.23, 0.82)	0.007 (0.004, 0.011)
Bulgaria	BGR	116	6.9 (3.8, 10.9)	11.2 (7.4, 15.4)	0.48 (0.26, 0.76)	0.007 (0.004, 0.011)
Australia	AUS	117	1.8 (1.0, 2.7)	8.6 (5.2, 12.7)	0.45 (0.26, 0.68)	0.007 (0.004, 0.010)
Sao Tome and Principe	STP	118	191.0 (26.3, 769.5)	25.0 (12.0, 42.7)	0.42 (0.06, 1.68)	0.006 (0.001, 0.024)
France	FRA	119	0.6 (0.2, 1.2)	12.2 (8.1, 16.3)	0.41 (0.15, 0.80)	0.006 (0.002, 0.011)
Paraguay	PRY	120	6.0 (3.3, 9.7)	14.3 (10.5, 18.5)	0.39 (0.22, 0.64)	0.006 (0.003, 0.009)
Guyana	GUY	121	46.8 (26.9, 71.5)	22.8 (16.4, 29.8)	0.37 (0.21, 0.57)	0.005 (0.003, 0.008)
Estonia	EST	122	25.5 (18.2, 33.7)	9.5 (6.8, 12.2)	0.34 (0.24, 0.45)	0.005 (0.004, 0.006)
Suriname	SUR	123	55.7 (32.9, 84.7)	17.5 (12.4, 23.6)	0.34 (0.20, 0.51)	0.005 (0.003, 0.007)
China, Hong Kong SAR	HKG	124	4.4 (2.9, 6.2)	7.3 (4.8, 9.9)	0.33 (0.22, 0.47)	0.005 (0.003, 0.006)
Singapore	SGP	125	5.5 (3.2, 8.1)	6.7 (4.1, 9.4)	0.33 (0.19, 0.48)	0.005 (0.003, 0.006)
Austria	AUT	126	3.6 (2.2, 5.5)	10.9 (7.3, 14.8)	0.32 (0.19, 0.49)	0.005 (0.003, 0.007)
Portugal	PRT	127	2.9 (1.6, 4.7)	16.1 (10.7, 22.2)	0.30 (0.16, 0.48)	0.004 (0.002, 0.007)
Hungary	HUN	128	2.6 (1.6, 3.9)	14.4 (9.6, 20.2)	0.26 (0.16, 0.38)	0.004 (0.002, 0.006)
Canada	CAN	129	0.6 (0.2, 1.1)	5.8 (3.1, 9.0)	0.21 (0.08, 0.40)	0.003 (0.001, 0.006)
Kiribati	KIR	130	163.2 (81.2, 273.8)	18.1 (12.6, 25.2)	0.21 (0.10, 0.35)	0.003 (0.002, 0.005)
Lebanon	LBN	131	3.6 (1.8, 6.0)	16.5 (9.8, 24.5)	0.20 (0.10, 0.34)	0.003 (0.002, 0.005)
Netherlands	NLD	132	1.1 (0.6, 1.7)	9.2 (4.9, 13.9)	0.19 (0.10, 0.30)	0.003 (0.002, 0.004)
Kuwait	KWT	133	4.3 (2.4, 7.0)	13.0 (7.5, 18.7)	0.19 (0.10, 0.30)	0.003 (0.002, 0.004)
Belgium	BEL	134	1.6 (0.8, 2.5)	7.9 (4.4, 11.9)	0.18 (0.09, 0.29)	0.003 (0.001, 0.004)
Cabo Verde	CPV	135	29.1 (3.8, 94.8)	24.4 (16.4, 32.8)	0.17 (0.02, 0.55)	0.002 (0.000, 0.008)
Marshall Islands	MHL	136	363.9 (125.2, 741.8)	24.8 (17.6, 34.6)	0.16 (0.05, 0.32)	0.002 (0.001, 0.005)
Qatar	QAT	137	5.7 (3.0, 9.1)	9.7 (5.7, 13.7)	0.16 (0.08, 0.25)	0.002 (0.001, 0.004)
Uruguay	URY	138	4.5 (2.5, 7.4)	13.6 (8.8, 19.7)	0.16 (0.09, 0.25)	0.002 (0.001, 0.004)
Fiji	FJI	139	16.0 (7.7, 28.0)	17.5 (11.2, 24.8)	0.15 (0.07, 0.26)	0.002 (0.001, 0.004)

Table S2: DALYs resulting from incident RR-TB in 2020, by country [continued]

Country	ISO3 code	Rank	RR-TB DALYs per 100,000	DALYs per RR-TB case	Total RR-TB DALYs (thousands)	Percent of global RR-TB burden (%)
Sweden	SWE	140	1.4 (0.8, 2.2)	10.3 (6.0, 15.5)	0.15 (0.08, 0.23)	0.002 (0.001, 0.003)
Serbia	SRB	141	1.9 (0.8, 3.3)	9.1 (6.1, 12.1)	0.14 (0.06, 0.24)	0.002 (0.001, 0.004)
Solomon Islands	SLB	142	18.2 (2.0, 56.1)	17.5 (11.0, 26.1)	0.13 (0.01, 0.39)	0.002 (0.000, 0.006)
Greece	GRC	143	1.1 (0.5, 2.1)	10.8 (7.5, 14.8)	0.12 (0.05, 0.22)	0.002 (0.001, 0.003)
China, Macao SAR	MAC	144	17.4 (9.7, 27.5)	11.2 (7.0, 16.7)	0.12 (0.07, 0.19)	0.002 (0.001, 0.003)
Israel	ISR	145	1.3 (0.7, 2.1)	8.6 (5.5, 12.3)	0.12 (0.06, 0.19)	0.002 (0.001, 0.003)
Czechia	CZE	146	1.1 (0.6, 1.7)	8.4 (4.8, 12.7)	0.11 (0.06, 0.18)	0.002 (0.001, 0.003)
Norway	NOR	147	2.0 (1.1, 3.2)	15.0 (9.5, 22.8)	0.11 (0.06, 0.17)	0.002 (0.001, 0.002)
Slovakia	SVK	148	1.9 (1.1, 3.1)	18.4 (12.6, 26.0)	0.10 (0.06, 0.17)	0.002 (0.001, 0.002)
Jordan	JOR	149	0.9 (0.3, 1.8)	6.9 (3.2, 10.9)	0.10 (0.03, 0.20)	0.001 (0.001, 0.003)
Comoros	COM	150	11.4 (1.9, 31.3)	19.8 (12.1, 29.8)	0.09 (0.02, 0.25)	0.001 (0.000, 0.004)
Trinidad and Tobago	TTO	151	5.5 (1.6, 11.7)	20.4 (14.1, 27.3)	0.08 (0.02, 0.18)	0.001 (0.000, 0.003)
Albania	ALB	152	2.9 (1.2, 5.2)	6.4 (3.5, 9.4)	0.08 (0.03, 0.15)	0.001 (0.001, 0.002)
Finland	FIN	153	1.4 (0.8, 2.2)	11.0 (7.2, 15.8)	0.08 (0.05, 0.12)	0.001 (0.001, 0.002)
Switzerland	CHE	154	0.9 (0.4, 1.6)	8.6 (5.0, 13.5)	0.08 (0.04, 0.14)	0.001 (0.001, 0.002)
Oman	OMN	155	1.7 (0.9, 2.8)	13.2 (7.8, 19.9)	0.08 (0.04, 0.13)	0.001 (0.001, 0.002)
Costa Rica	CRI	156	1.5 (0.7, 2.7)	10.3 (6.6, 14.9)	0.07 (0.04, 0.14)	0.001 (0.001, 0.002)
Vanuatu	VUT	157	20.7 (6.8, 43.1)	20.5 (13.0, 31.1)	0.06 (0.02, 0.13)	0.001 (0.000, 0.002)
French Polynesia	PYF	158	18.2 (7.8, 32.8)	12.2 (7.6, 17.8)	0.05 (0.02, 0.10)	0.001 (0.000, 0.001)
Bahrain	BHR	159	3.7 (1.6, 6.9)	11.3 (6.8, 18.1)	0.05 (0.02, 0.10)	0.001 (0.000, 0.001)
New Zealand	NZL	160	1.0 (0.2, 2.3)	7.8 (4.3, 11.4)	0.05 (0.01, 0.11)	0.001 (0.000, 0.002)
Ireland	IRL	161	0.9 (0.4, 1.8)	9.1 (5.5, 13.7)	0.05 (0.02, 0.09)	0.001 (0.000, 0.001)
Denmark	DNK	162	0.7 (0.3, 1.3)	8.5 (5.0, 12.1)	0.04 (0.02, 0.07)	0.001 (0.000, 0.001)
Tuvalu	TUV	163	353.9 (109.9, 735.8)	22.3 (16.0, 29.9)	0.04 (0.01, 0.08)	0.001 (0.000, 0.001)
Bosnia and Herzegovina	BIH	164	1.1 (0.6, 2.1)	8.4 (5.8, 11.0)	0.04 (0.02, 0.07)	0.001 (0.000, 0.001)
Mauritius	MUS	165	2.7 (1.2, 5.0)	17.3 (11.9, 24.4)	0.03 (0.02, 0.07)	0.001 (0.000, 0.001)
North Macedonia	MKD	166	1.5 (0.7, 2.8)	10.9 (7.1, 14.8)	0.03 (0.02, 0.06)	0.000 (0.000, 0.001)
Bahamas	BHS	167	7.3 (3.1, 14.0)	15.2 (9.2, 22.7)	0.03 (0.01, 0.06)	0.000 (0.000, 0.001)

Table S2: DALYs resulting from incident RR-TB in 2020, by country [continued]

Country	ISO3 code	Rank	RR-TB DALYs per 100,000	DALYs per RR-TB case	Total RR-TB DALYs (thousands)	Percent of global RR-TB burden (%)
Croatia	HRV	168	0.7 (0.3, 1.4)	20.4 (16.7, 24.5)	0.03 (0.01, 0.06)	0.000 (0.000, 0.001)
Dominica	DMA	169	36.9 (4.0, 113.3)	20.6 (15.3, 26.7)	0.03 (0.00, 0.08)	0.000 (0.000, 0.001)
Belize	BLZ	170	6.2 (2.2, 12.8)	20.5 (13.5, 27.8)	0.02 (0.01, 0.05)	0.000 (0.000, 0.001)
Guam	GUM	171	13.5 (5.8, 25.7)	11.9 (7.4, 17.4)	0.02 (0.01, 0.04)	0.000 (0.000, 0.001)
Brunei Darussalam	BRN	172	4.9 (2.0, 9.6)	12.7 (8.8, 18.0)	0.02 (0.01, 0.04)	0.000 (0.000, 0.001)
Greenland	GRL	173	37.8 (18.7, 61.5)	14.8 (10.0, 20.2)	0.02 (0.01, 0.03)	0.000 (0.000, 0.000)
Micronesia (Federated States of)	FSM	174	15.9 (2.0, 44.9)	22.1 (13.3, 31.8)	0.02 (0.00, 0.05)	0.000 (0.000, 0.001)
Northern Mariana Islands	MNP	175	34.6 (10.6, 72.5)	15.9 (10.3, 21.8)	0.02 (0.01, 0.04)	0.000 (0.000, 0.001)
United Arab Emirates	ARE	176	0.2 (0.0, 0.5)	11.0 (6.0, 16.9)	0.02 (0.00, 0.05)	0.000 (0.000, 0.001)
Maldives	MDV	177	3.0 (0.4, 8.3)	9.5 (6.4, 12.7)	0.02 (0.00, 0.04)	0.000 (0.000, 0.001)
Malta	MLT	178	2.8 (0.2, 8.7)	13.7 (7.8, 22.0)	0.01 (0.00, 0.04)	0.000 (0.000, 0.001)
Jamaica	JAM	179	0.5 (0.1, 1.4)	20.7 (13.1, 29.8)	0.01 (0.00, 0.04)	0.000 (0.000, 0.001)
Puerto Rico	PRI	180	0.4 (0.2, 0.9)	16.4 (11.2, 22.2)	0.01 (0.01, 0.03)	0.000 (0.000, 0.000)
Palestinian Territory	PSE	181	0.3 (0.0, 0.9)	23.7 (12.8, 34.7)	0.01 (0.00, 0.04)	0.000 (0.000, 0.001)
Palau	PLW	182	42.0 (12.3, 92.4)	17.3 (10.9, 24.3)	0.01 (0.00, 0.02)	0.000 (0.000, 0.000)
Cyprus	CYP	183	0.6 (0.1, 1.5)	10.1 (6.9, 13.8)	0.01 (0.00, 0.02)	0.000 (0.000, 0.000)
Slovenia	SVN	184	0.3 (0.1, 0.6)	11.6 (7.6, 16.6)	0.01 (0.00, 0.01)	0.000 (0.000, 0.000)
Samoa	WSM	185	2.6 (0.5, 6.0)	15.6 (8.9, 23.3)	0.01 (0.00, 0.01)	0.000 (0.000, 0.000)
Nauru	NRU	186	44.6 (2.1, 151.8)	13.1 (9.2, 17.4)	0.01 (0.00, 0.02)	0.000 (0.000, 0.000)
Curaçao	CUW	187	2.6 (0.4, 7.0)	10.6 (6.3, 16.1)	0.00 (0.00, 0.01)	0.000 (0.000, 0.000)
Montenegro	MNE	188	0.8 (0.2, 1.6)	5.8 (3.0, 8.7)	0.00 (0.00, 0.01)	0.000 (0.000, 0.000)
Luxembourg	LUX	189	0.7 (0.1, 2.0)	10.8 (6.0, 17.3)	0.00 (0.00, 0.01)	0.000 (0.000, 0.000)
New Caledonia	NCL	190	1.5 (0.4, 3.1)	9.9 (5.9, 14.2)	0.00 (0.00, 0.01)	0.000 (0.000, 0.000)
Iceland	ISL	191	0.4 (0.0, 1.3)	14.8 (10.8, 19.2)	0.00 (0.00, 0.00)	0.000 (0.000, 0.000)
Seychelles	SYC	192	0.9 (0.1, 2.8)	12.3 (6.3, 20.6)	0.00 (0.00, 0.00)	0.000 (0.000, 0.000)

DALYs = disability-adjusted life years. RR-TB = rifampicin-resistant tuberculosis. Values in parentheses represent 95% uncertainty intervals.

Table S3: Partial rank correlation coefficients (PRCCs) between model parameters and the total global DALYs due to RR-TB in 2020.

Parameter	Rank	PRCC
Odds ratio of chronic post-TB respiratory disease for RR-TB	1	0.92 (0.89, 0.94)
Slope term for log-linear model for OR of chronic respiratory disease in post-TB	2	0.83 (0.79, 0.86)
Prevalence of rifampicin resistance in new tuberculosis cases (global average)	3	0.66 (0.62, 0.71)
TB incidence (global total)	4	0.54 (0.49, 0.59)
Weight for calculating tuberculosis case fatality for RR-TB treated with a first-line regimen	5	-0.51 (-0.56, -0.46)
TB case fatality (global average)	6	0.47 (0.41, 0.52)
Linear term for log-linear model for mortality by FEV1%	7	0.44 (0.39, 0.50)
Intercept term for log-linear model for OR of chronic respiratory disease with post-TB	8	-0.39 (-0.45, -0.33)
Disability weight for moderate COPD	9	0.38 (0.33, 0.44)
Prevalence of rifampicin resistance in previously treated tuberculosis cases (global average)	10	0.31 (0.25, 0.37)
Disability weight for severe COPD	11	0.25 (0.18, 0.31)
Log odds ratio of RR-TB for age 15+ vs. age 0-14 years (global average)	12	-0.24 (-0.30, -0.18)
Quadratic term for log-linear model for mortality by FEV1%	13	0.23 (0.17, 0.29)
Mortality rate ratio for post-TB individuals	14	0.18 (0.11, 0.24)
Disability weight for tuberculosis	15	0.15 (0.09, 0.21)
Duration of untreated tuberculosis	16	0.14 (0.08, 0.20)
Odds ratio of tuberculosis case fatality for RR-TB on second-line regimen (global average)	17	0.14 (0.08, 0.20)
Duration of treated tuberculosis	18	0.12 (0.06, 0.19)
Odds ratio of RR-TB for HIV infected vs. HIV uninfected (global average)	19	0.12 (0.06, 0.18)
Probability of RR-TB diagnosis among individuals diagnosed with tuberculosis (global average)	20	0.08 (0.02, 0.14)
Odds ratio of tuberculosis case fatality for individuals not receiving tuberculosis treatment	21	0.07 (0.01, 0.13)
Disability weight for HIV on ART	22	-0.04 (-0.10, 0.03)
Odds ratio of tuberculosis case fatality for HIV infected not on ART	23	0.03 (-0.03, 0.09)
Odds ratio of RR-TB for males vs. females (global average)	24	-0.03 (-0.09, 0.03)
Disability weight for tuberculosis and HIV	25	-0.03 (-0.09, 0.04)
Odds ratio of tuberculosis case fatality for HIV infected on ART	26	0.02 (-0.04, 0.08)
Duration of treated tuberculosis, with HIV	27	-0.02 (-0.08, 0.04)
Duration of untreated tuberculosis, with HIV	28	-0.01 (-0.07, 0.05)
Probability of second-line treatment among individuals diagnosed with RR-TB (global average)	29	-0.01 (-0.07, 0.05)
Disability weight for symptomatic HIV, no ART	30	0.00 (-0.06, 0.07)
Disability weight for mild COPD	31	0.00 (-0.06, 0.07)

DALYs = disability-adjusted life years. RR-TB = rifampicin-resistant tuberculosis. Values in parentheses represent 95% uncertainty intervals.

Table S4: Global RR-TB burden of disease results for alternative analytic specifications, as compared to the main analysis.

Specification	Outcome*	Total RR-TB DALYs (millions)	DALYs per RR-TB case	Percent of total TB burden (%)
Main analysis	Value	6.93 (5.52, 8.53)	17.3 (13.8, 20.6)	5.4 (4.4, 6.3)
	Percent increase	*Reference*	*Reference*	*Reference*
Duration of disease associated with RR-TB assumed to be 50% greater than for RS-TB	Value	7.13 (5.70, 8.75)	17.7 (14.1, 21.1)	5.5 (4.5, 6.5)
	Percent increase	2.8 (1.4, 4.9)	2.8 (1.4, 4.9)	2.7 (1.4, 4.7)
Case fatality for RR-TB treated with first-line regimen assumed to be same as untreated individuals	Value	7.25 (5.83, 8.78)	18.0 (14.7, 21.2)	5.6 (4.7, 6.6)
	Percent increase	4.7 (1.0, 10.5)	4.7 (1.0, 10.5)	4.7 (1.0, 10.6)
Case fatality for RR-TB treated with first-line regimen assumed to be same as with second-line regimen	Value	6.44 (4.98, 8.06)	16.0 (12.6, 19.3)	5.0 (4.1, 5.9)
	Percent increase	-7.1 (-12.0, -2.6)	-7.1 (-12.0, -2.6)	-7.2 (-12.1, -2.7)
Case fatality for untreated RR-TB assumed to be greater than for RS-TB (OR = 1.5)	Value	7.49 (6.07, 9.05)	18.6 (15.2, 21.8)	5.8 (4.8, 6.8)
	Percent increase	8.1 (5.9, 11.0)	8.1 (5.9, 11.0)	8.1 (5.9, 11.0)
Post-TB mortality rates based on estimates reported by Lee Rodriguez (2020)	Value	5.80 (4.99, 6.66)	14.4 (12.6, 16.2)	5.0 (4.4, 5.6)
	Percent increase	-15.8 (-25.5, -3.2)	-15.8 (-25.5, -3.2)	-6.9 (-15.5, 2.7)
Post-TB mortality rates and disability weights for RR-TB the same as for RS-TB	Value	5.86 (4.91, 6.95)	14.6 (12.4, 17.0)	4.6 (4.1, 5.1)
	Percent increase	-15.0 (-24.9, -1.4)	-15.0 (-24.9, -1.4)	-14.3 (-23.8, -1.3)

DALYs = disability-adjusted life years. RR-TB = rifampicin-resistant tuberculosis. Values in parentheses represent 95% uncertainty intervals. * Results given as "Percent difference" represent the difference between the value estimated in the sensitivity analysis and the value in the main analysis, expressed as a percentage of the main analysis value.

Figure S1: Data availability by country and analytic input.

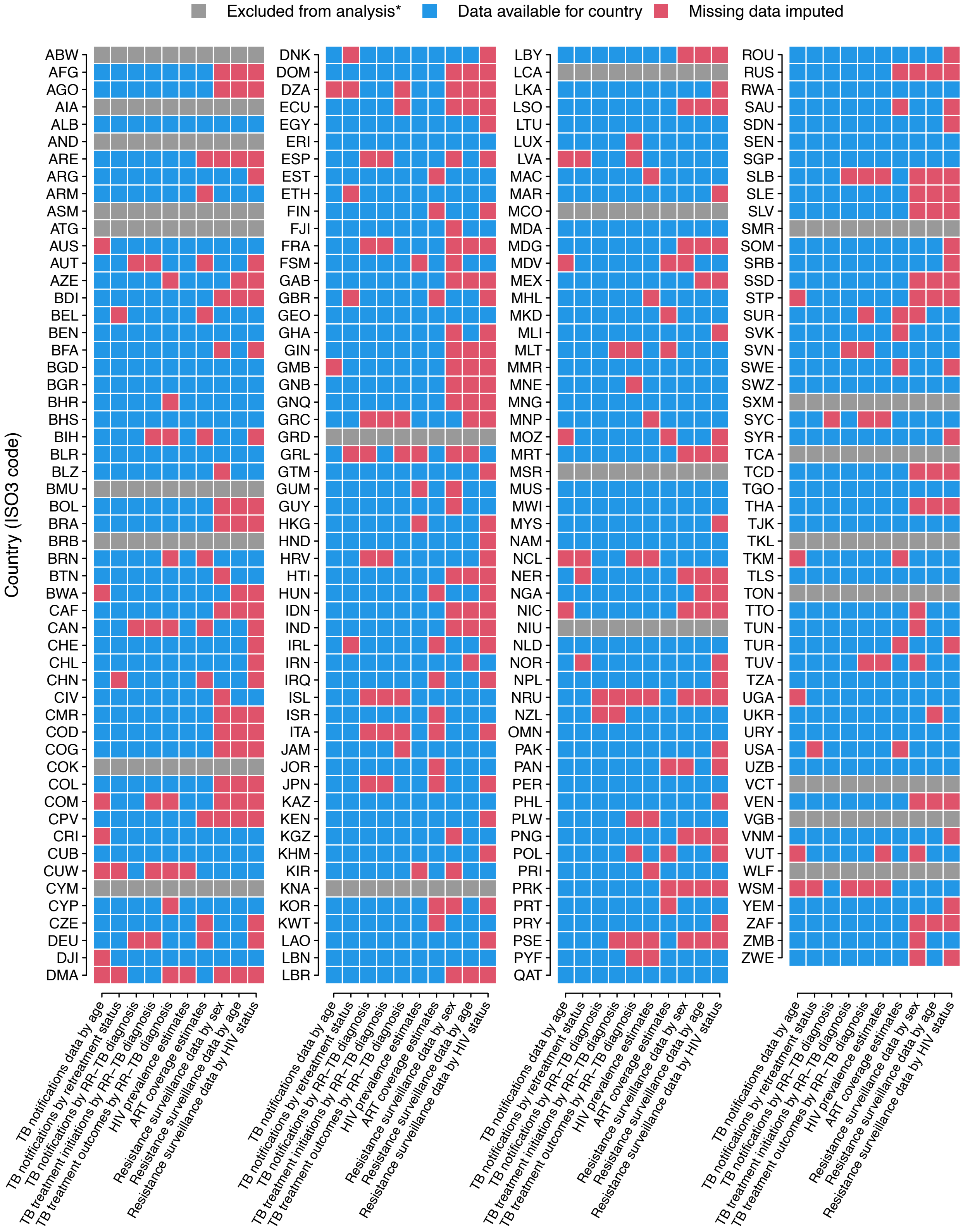

*Countries with less than 10 estimated TB cases for 2020 were excluded from analysis.

Supplementary methods

We constructed a hypothetical cohort representing the global population who developed tuberculosis disease in 2020, stratified by country, age, sex, HIV status, RR-TB status, treatment status, and survival of the disease episode. We used these estimates to populate a compartmental model, stratified by these same dimensions, which was used to project health outcomes during the TB disease episode and over the remaining lifetime of surviving individuals, with an annual timestep.

Number and distribution of individuals developing tuberculosis in 2020

Estimates of the total number of individuals developing tuberculosis in 2020 were extracted from epidemiological estimates produced by the WHO Global Tuberculosis Programme.[1] These are stratified by country, sex, and age group (0-4, 5-14, 15-24, 25-34, 35-44, 45-54, 55-64, 65 plus). For each country and sex, we interpolated these reported values to obtain incidence estimates by single year of age. To do so we specified a smooth function for the incidence rate (10-parameter cubic b-spline with a first-order difference penalty), constrained to reproduce the age-group-stratified WHO values. To account for uncertainty in incidence estimates we constructed probability distributions for country-level incidence matching the uncertainty intervals reported by WHO, and scaled incidence within each age/sex stratum proportionally.

The fraction of incident TB cases receiving treatment (including individuals with RR-TB receiving an inappropriate 1st line regimen) was based on the number of notified tuberculosis cases within each country, sex, and age group for 2020 (as reported to the WHO Global Tuberculosis Programme[1]), divided by estimated incidence. Interpolation, via a 10-parameter cubic b-spline with a first-order difference penalty, was used to estimate the fraction treated by single year of age, constrained to assume that this fraction was less than 99% within any individual age stratum. For countries with missing notification data we assumed that WHO-estimated treatment coverage applied to all ages uniformly. We removed countries with less than 10 estimated cases for 2020. Applying these criteria retained 192 countries, representing 9.9 million tuberculosis cases, 99.99% of all cases globally. Figure S2 shows point estimates for the fraction treated for each of these 192 countries.

Figure S2: Fraction of study cohort receiving tuberculosis treatment, by country*.

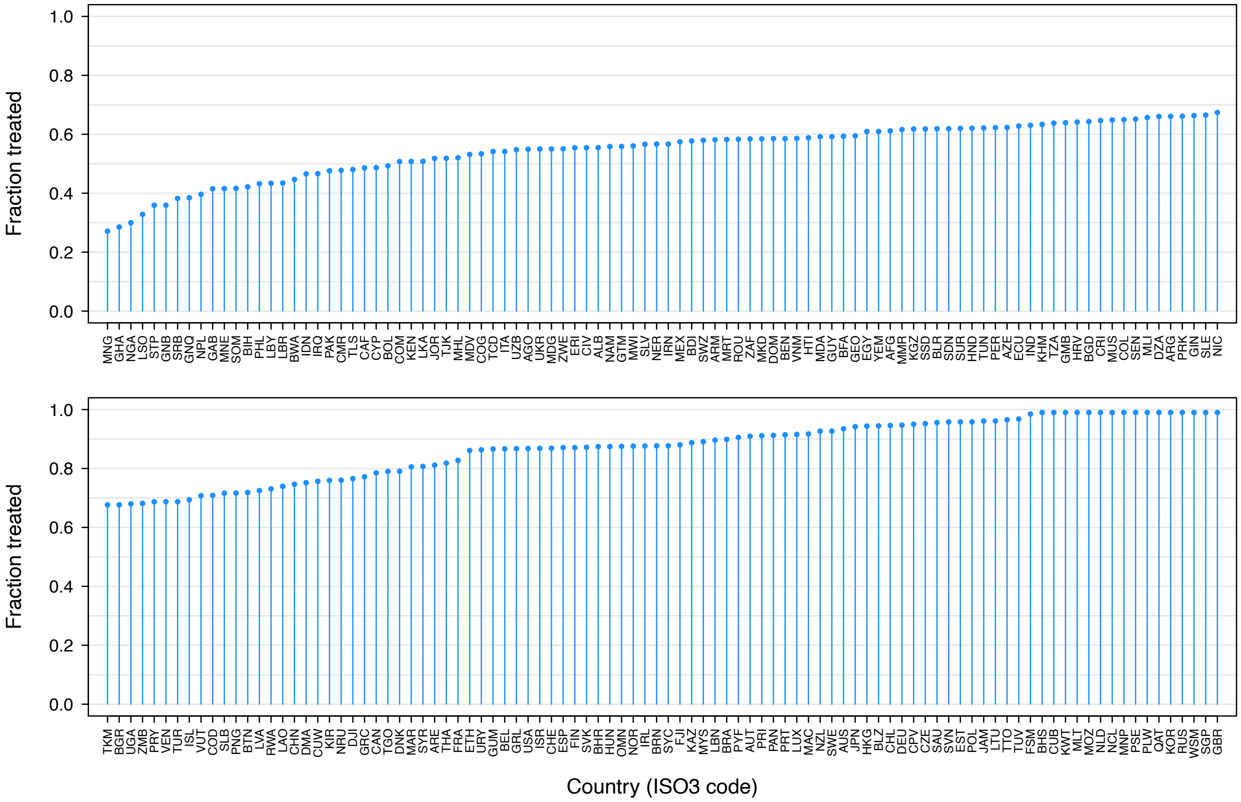

* Countries ordered by fraction treated, from left of upper panel to right of lower panel.

To stratify cases by HIV status, we multiplied the odds of HIV in each age group in the general population (based on UNAIDS epidemiological estimates[10]) by a common odds ratio calibrated to reproduce the overall number of TB-HIV cases in each country based on WHO Global Tuberculosis Programme estimates.[1] For countries for which this value was missing we assumed TB-HIV prevalence was 0%. Within each age and sex stratum, the fraction treated was assumed to be independent of HIV status. Figure S3 shows the fraction of the study cohort with HIV, for each modelled country.

Figure S3: Fraction of study cohort coinfected with HIV, by country*.

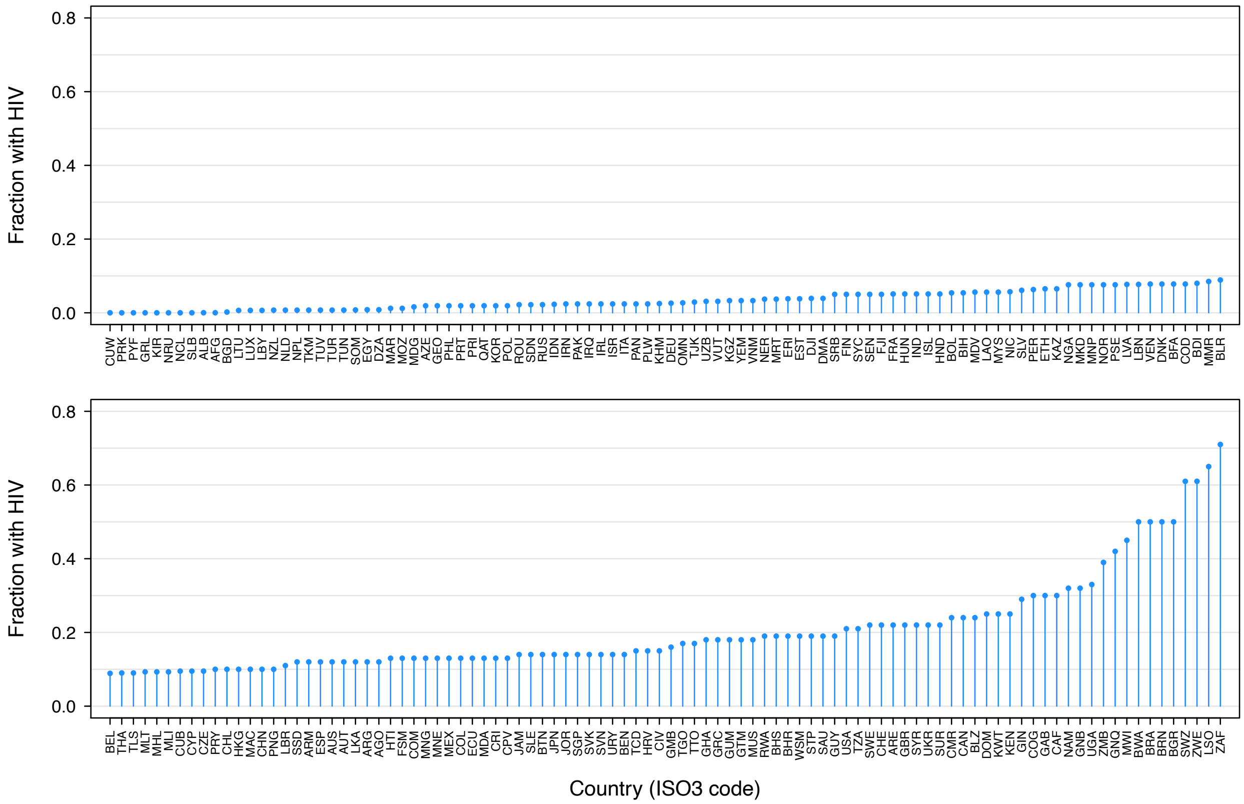

* Countries ordered by fraction with HIV, from left of upper panel to right of lower panel.

To stratify this cohort by RR-TB status, we first calculated the fraction of incident tuberculosis cases with rifampicin resistance for each country. We took WHO Global Tuberculosis Programme estimates of the prevalence of rifampicin resistance among new cases and previously treated cases,[1] and multiplied these values by the fraction of tuberculosis cases that are new and previously treated (respectively), and summed these two values. This produced estimates of the fraction of all incident tuberculosis cases with rifampicin resistance, by country (Figure S4).

Figure S4: Fraction of study cohort with rifampicin resistance, by country*.

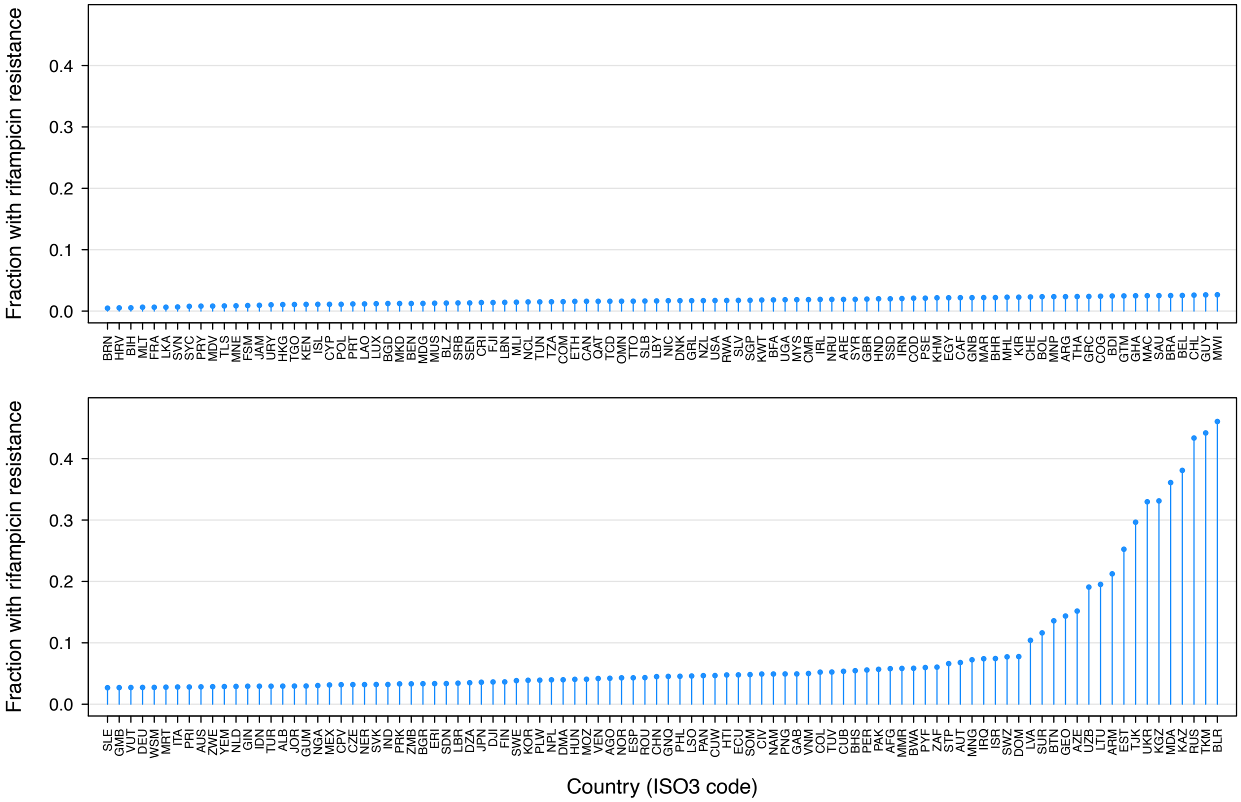

* Countries ordered by fraction with rifampicin resistance, from left of upper panel to right of lower panel.

We used data from WHO tuberculosis drug resistance surveys and routine surveillance activities to decompose the rifampicin-resistant stratum by sex, age, and HIV status.[11] These data provide separate tabulations of drug resistance by sex, HIV status, and age (0-15 vs. 15+ years-old). After excluding surveys with low completeness of required variables (<90% completeness) and surveys without national coverage, we estimated logistic regression models for the relative prevalence of RR-TB among males vs. females (378 survey-years, covering 127 countries), HIV infected vs. HIV uninfected individuals (317 survey-years, covering 98 countries), and individuals aged over 15 years vs. individuals aged 0-15 years (793 survey-years, covering 148 countries). These regressions included fixed effects for WHO region (with the European Region additionally stratified by Former Soviet Union status, due to the different epidemiology of tuberculosis drug resistance in these countries), and random effects for each country represented in the data. We used regional averages to impute values for countries not represented in the dataset (70 countries for sex, 104 countries for HIV status, and 55 countries for age), and assumed the estimated odds ratios were independent (i.e. the odds ratio estimated for one factor do not vary across levels of the other two factors). Figure S5 shows the distribution of country-level estimates for the odds ratios of rifampicin resistance by sex, age, and HIV status, by WHO region. To calculate the prevalence of RR-TB in each age/sex/HIV stratum, we calibrated the odds of RR-TB in the reference group (female, 0-14 years old, HIV uninfected) so that once all odds ratios were applied the overall country-level prevalence of rifampicin resistance matched the values shown in Figure S4.

Figure S5: Distribution of country-specific odds ratios of rifampicin resistance for males vs. females (Panel A), HIV-infected vs. non-infected (Panel B), and age 15+ years old vs. age 0-15 years (Panel C), by WHO region.

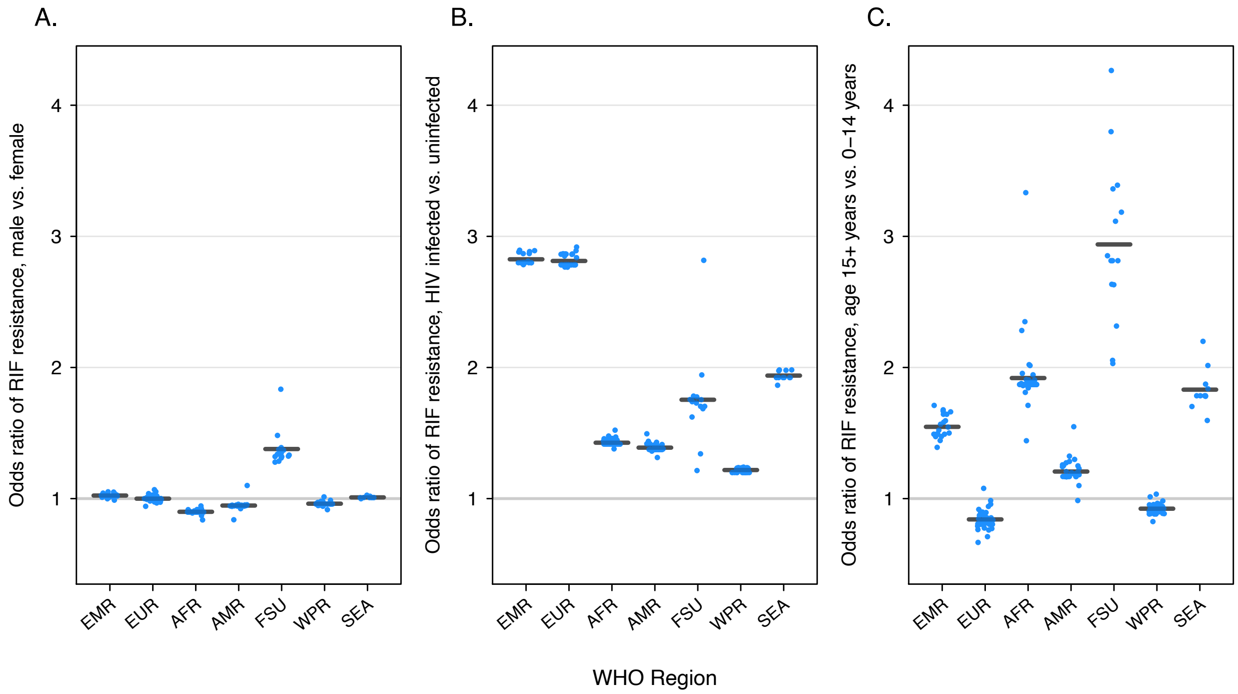

Former Soviet Union countries reported separately from the European Region given their different epidemiology of tuberculosis drug resistance. EMR = Eastern Mediterranean Region, EUR = European Region, excluding Former Soviet Union countries, AFR = African Region, AMR = Region of the Americas, FSU = Former Soviet Union, WPR = Western Pacific Region, SEA = Southeast Asia Region. Points represent individual country values, bars represent regional averages. Data used for this analysis include both new and previously treated cases.

We subdivided the RR-TB population into four diagnosis and treatment categories: (A) individuals not receiving tuberculosis diagnosis and treatment, (B) individuals diagnosed with tuberculosis but who failed to receive a RR-TB diagnosis, (C) individuals diagnosed with RR-TB who failed to receive a second-line treatment regimen, and (D) individuals diagnosed with RR-TB and initiated on a second-line treatment regimen. Individuals in category A were assumed to receive no treatment, individuals in categories B and C were assumed to receive a first-line tuberculosis treatment regimen (inappropriate for their drug resistance profile), and individuals in category D were assumed to receive a second-line treatment regimen.

The fraction not receiving tuberculosis diagnosis and treatment (category A) was assumed to be the same for RS-TB and RR-TB, and based on the values shown in Figure S2. To calculate the fraction of RR-TB individuals diagnosed with tuberculosis whose rifampicin resistance was identified (categories C+D divided by B+C+D) we collated country-reported data on the number of MDR-TB/RR-TB diagnoses and compared this to number of individuals with RR-TB receiving a tuberculosis diagnosis. We calculated this value as the product of RR-TB incidence multiplied by the ratio of overall tuberculosis diagnoses to total tuberculosis incidence, under the assumption that individuals with RR-TB have the same probability of tuberculosis diagnosis as individuals with RS-TB. We estimated the fraction of individuals with RR-TB receiving a RR-TB diagnosis among those diagnosed with tuberculosis from a logistic regression fit to grouped data (by country and year) on country-reported MDR-TB/RR-TB diagnoses and our estimated values for total RR-TB receiving a tuberculosis diagnosis, as described above. We fit this model to data from 2017 to 2019 (most recent 3 years with available data) to reduce sampling uncertainty. We included fixed effects for region and year, and random effects for country. This approach produced estimates of the fraction of individuals with RR-TB receiving a RR-TB diagnosis, among those diagnosed with tuberculosis. We used regional averages to impute values for countries with missing data (14 countries). Figure S6A shows the distribution of these country-level estimates by WHO region. We took a similar regression approach to estimate the

fraction of individuals with diagnosed RR-TB who were initiated on a second-line regimen (category D divided by C+D), comparing country-reported data on the number of MDR-T/RR-TB diagnoses and the number of individuals initiating second-line treatment regimens. We fit logistic regression models using data for 2017 to 2019, with fixed effects for region and year, and random effects for country. Values for countries with missing data were imputed using regional averages (20 countries). Figure S6B shows the distribution of these country-level estimates by WHO region.

Figure S6: Estimated fraction of individuals diagnosed with tuberculosis who receive a RR-TB diagnosis (Panel A), estimated fraction of individuals diagnosed with RR-TB who receive a second-line treatment regimen (Panel B), and estimated odds ratio of surviving tuberculosis treatment, for second-line vs. first-line regimens (Panel C), by WHO region.

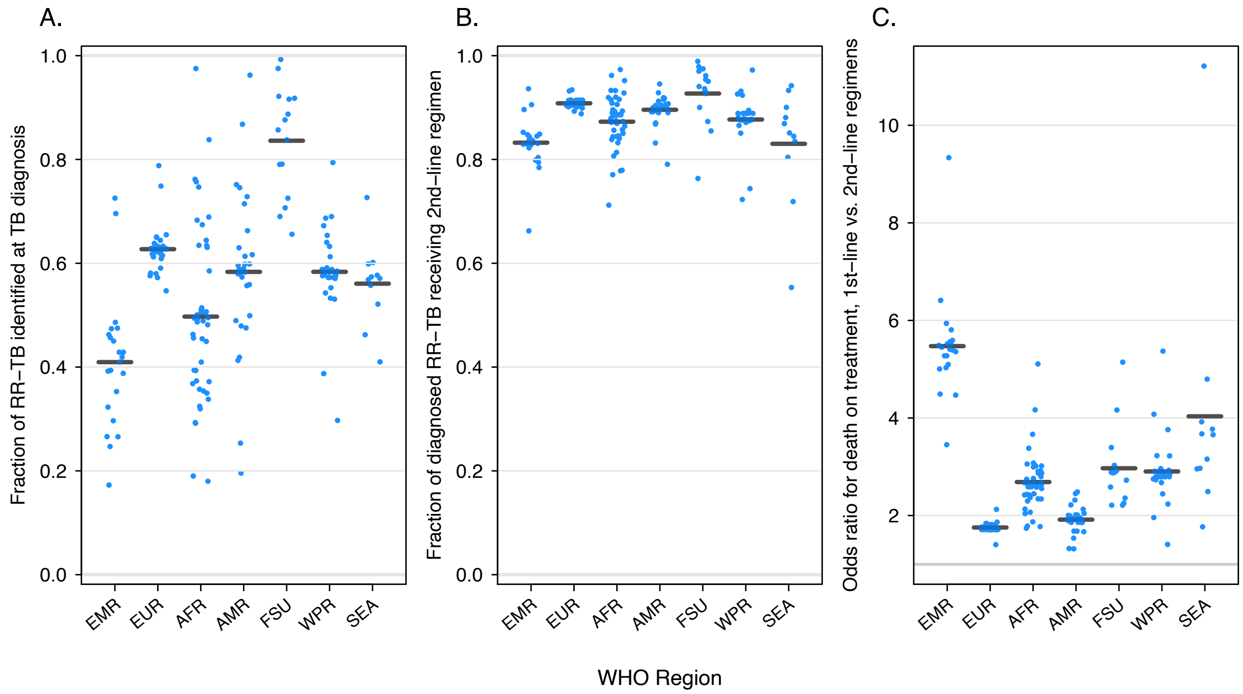

Former Soviet Union countries reported separately from the European Region given their different epidemiology of tuberculosis drug resistance. EMR = Eastern Mediterranean Region, EUR = European Region, excluding Former Soviet Union countries, AFR = African Region, AMR = Region of the Americas, FSU = Former Soviet Union, WPR = Western Pacific Region, SEA = Southeast Asia Region. Points represent individual country values, bars represent regional averages. Data on second-line regimens are assumed to represent RR-TB treated with an appropriate regimen. Data on first-line regimens are assumed to represent RS-TB treated with an appropriate regimen.

Duration and disability of tuberculosis disease episode

We based assumptions around the duration of disability during the tuberculosis disease episode on values estimated by the WHO, stratified by treatment and HIV status (Table S1).[4] In the main analysis we assumed the duration of disease was the same for RS-TB and RR-RB, and examined alternative assumptions in sensitivity analyses.

The disability weight for tuberculosis disease was based on current Global Burden of Disease estimates.[3] For HIV-uninfected individuals with tuberculosis disease, this value is 0.333. For HIV-infected individuals with tuberculosis disease this value is 0.408. For HIV-uninfected individuals without tuberculosis we assumed a disability weight of zero. For HIV-infected individuals without tuberculosis we averaged the disability weights for ‘HIV: symptomatic, pre-AIDS’ (0.274) and ‘HIV/AIDS: receiving antiretroviral treatment’ (0.078), weighted by the fraction of HIV-infected individuals receiving antiretroviral treatment in each country, as reported by UNAIDS for 2020.[10] We calculated the incremental disability weight associated with tuberculosis disease as the difference between individuals with and without tuberculosis disease, stratified by HIV status.

Fraction surviving the tuberculosis disease episode

To estimate the fraction surviving the tuberculosis disease episode we specified odds ratios describing differences in survival probabilities by age, HIV, and tuberculosis treatment status. We estimated mortality odds ratios by age (Figure S7) using detailed data on case fatality among notified tuberculosis cases in Brazil (data for Brazil were used as detailed data of this type are not widely available).[12]

Figure S7: Odds ratios of death during the tuberculosis episode, by age*.

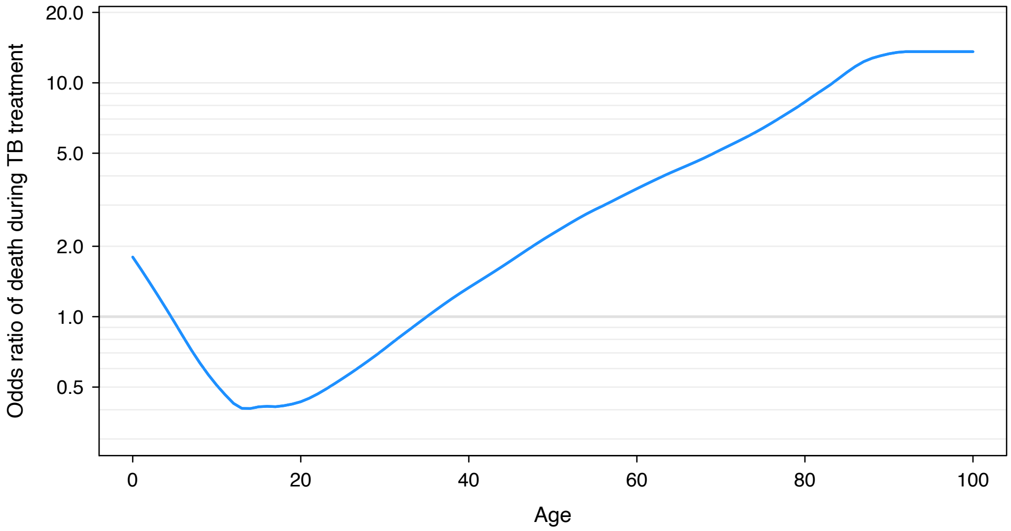

*Odds ratios normalized to a value of 1.0 for 35-year-old individuals.

Odds ratios of mortality for HIV infected individuals (OR = 3.66 for HIV infected not on ART, and 1.54 for HIV infected on ART, as compared to HIV uninfected individuals), and for individuals not receiving tuberculosis treatment (OR = 28.3, as compared to treated individuals), were based on mortality risks used in WHO Global Tuberculosis Programme epidemiological estimations.[4] In addition, we allowed for difference in survival between individuals with RS-TB receiving a first-line regimen (assumed to be all RS-TB individuals), individuals with RR-TB receiving a second-line regimen, and individuals with RR-TB inappropriately receiving a first-line regimen. To calculate mortality odds ratios for RR-TB on second-line treatment vs. RS-TB on first-line treatment, we fit a logistic regression model to country-reported data on cohort size and deaths on treatment for first and second-line regimens, pooling data across 2017 to 2019 (most recent 3 years with available data) to reduce sampling uncertainty. We included fixed effects for region and year, and random effects for country. We used regional averages to impute values for countries with missing data (32 countries). Figure S6C shows the distribution of these estimates by WHO region.

For individuals with RR-TB inappropriately receiving a first-line regimen, we assumed mortality rates for this group were higher than for individuals with RR-TB receiving a second-line regimen, but lower than untreated individuals. We operationalized this by calculating the mortality rate for individuals with RR-TB receiving a first-line regimen as a weighted average of these two extremes. The parameter defining this weighting was given a Beta(3,3) prior, which produces a mean value of 0.5 and a 95% interval of 0.15-0.85, and we tested extreme values of 0.0 and 1.0 in sensitivity analysis. Individuals with RR-TB and RS-TB not receiving tuberculosis treatment were assumed to have the same mortality risks. We applied the mortality odds ratios to each country, and calibrated overall mortality risks to reproduce the country-specific case fatality rate reported by the WHO Global Tuberculosis Programme for 2020 (Figure S8).[1]

Figure S8: Estimated fraction of individuals dying during the tuberculosis disease episode, by country*.

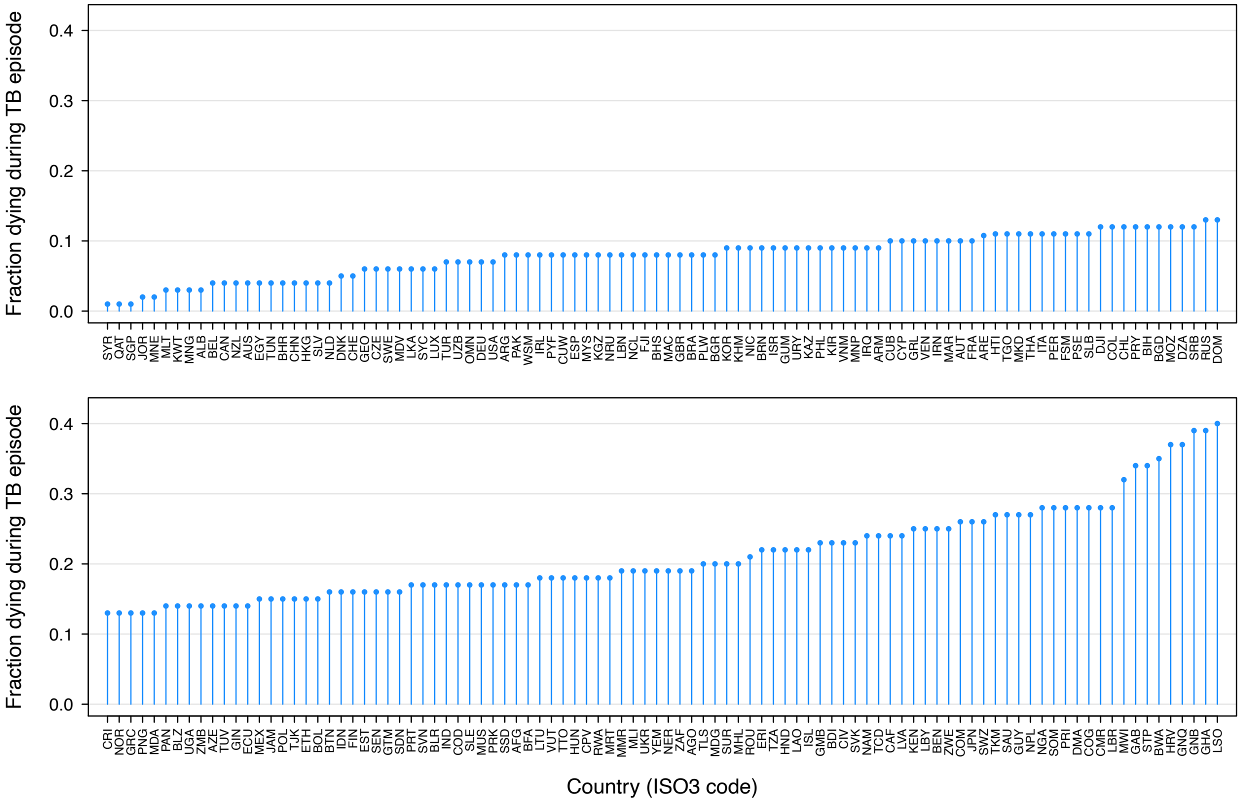

* Countries ordered by fraction dying during the TB episode, from left of upper panel to right of lower panel.

Future mortality risks for individuals surviving tuberculosis disease

Individuals surviving tuberculosis disease face elevated mortality risks compared to the general population. In a recent meta-analysis of ten cohorts receiving tuberculosis treatment, the standardized mortality ratio was 2.91 (95% interval 2.21–3.84) when compared to individuals without tuberculosis.[6] These elevated risks reflect a combination of (i) the causal impact of tuberculosis disease on future mortality risks, and (ii) individual characteristics that are correlated with both mortality rates and tuberculosis disease (Figure S9). Estimating the causal impact of tuberculosis disease on survival requires decomposing these two effects.

Figure S9: Assumed causal and non-causal relationships linking tuberculosis disease to future health outcomes.

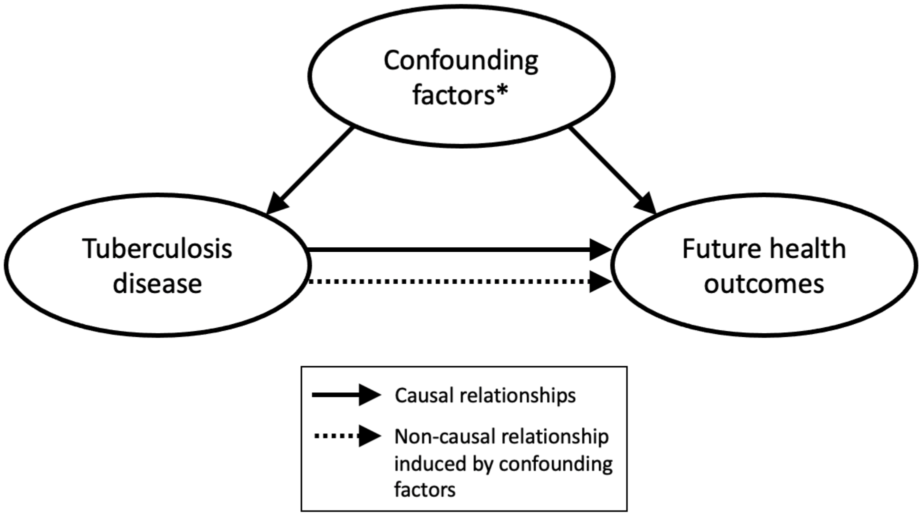

* Confounding factors will include socioeconomic status, living conditions, comorbid health conditions, and health behaviors that influence risks of tuberculosis acquisition and progression, and separately influence future health outcomes. The goal of this analysis is to estimate the effect on future health outcomes causally attributable to tuberculosis disease, and omit the non-causal component (dashed line).

To estimate the causal impact of tuberculosis disease on future health outcomes we assumed that the effects of tuberculosis on subsequent mortality can be approximated by the effects of tuberculosis on lung function, and the resulting effects of reduced lung function on mortality rates. While this approach only captures one of several mechanisms through which tuberculosis may impact future mortality risks, it is likely the major contribution to these risks, and provides a more conservative and defensible estimation strategy.

We first estimated future all-cause general population mortality rates for each country, sex, year of age, and calendar year, by interpolating UN Population Division abridged life tables. For HIV infected individuals, we assumed a life expectancy that is 8 (6-10) years shorter compared to HIV-negative individuals, calculated the mortality rate ratio which would produce this shortened lifespan, and applied this mortality rate ratios to all HIV infected individuals.

Secondly, we assumed that the published SMR (standardized mortality ratio) from the Romanowski study[6] could be interpreted as a mortality rate ratio for individuals with post-TB compared to the general population, conditional on age, sex, and country. To calculate future mortality rates for individuals surviving tuberculosis disease in 2019, we multiplied the all-cause general population mortality rate for each stratum by 2.91.

To estimate the *causal* impact of tuberculosis disease on future health we first estimated the additional COPD among individuals surviving tuberculosis disease. Two systematic reviews have assessed the elevated COPD rates among individuals surviving tuberculosis,[9,13] and the more recent of these[9] described a relationship between the odds ratio of COPD among post-TB individuals and country-level incidence. In this relationship, country-level incidence is likely a proxy for the extent of lung damage that individuals with tuberculosis disease experience before tuberculosis cure, due to delays in case detection and treatment initiation. We estimated a meta-regression model to summarize this relationship (Figure S10) and used the results from this analysis to estimate an odds ratio of COPD among post-TB individuals for each country in the analysis, based on their estimated tuberculosis incidence for 2020.

Figure S10: Published estimates for the adjusted odds ratio of COPD among individuals surviving tuberculosis, compared to a matched control group, with results from a meta-regression fit to these study estimates.

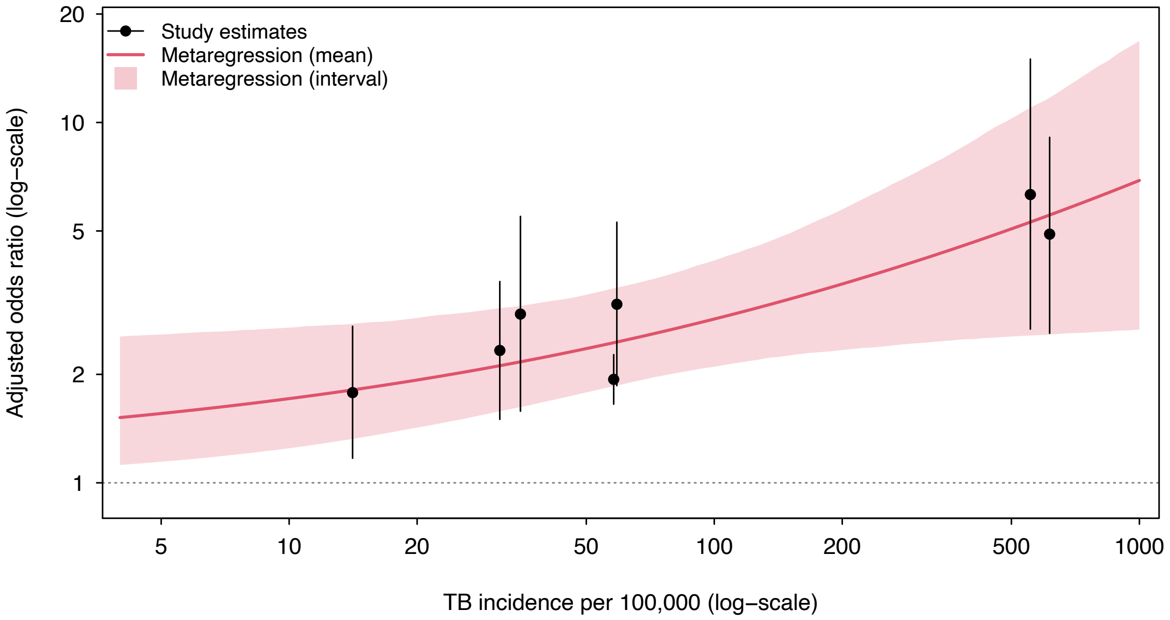

We used the results of Duong *et al* 2019[7] to describe the population distribution of lung function (quantified as FEV1%), and assumed that tuberculosis would result in a downward shift of this distribution. For each country, the reduction in FEV1% among post-TB individuals was estimated to match the odds ratios described above, with FEV1% <80% used as the threshold for COPD (consistent with classes 2-4 in the GOLD system for categorizing COPD). We fit a quadratic function to the mortality rate ratios reported by Duong *et al* 2019 to produce a relationship between FEV1% and mortality, and estimated the mortality risk ratio for post-TB as the average mortality rate based on the post-TB FEV1% distribution compared to the distribution without post-TB. This approach produced country-level mortality rate ratios that varied between 1.02 and 1.33, with a median of 1.14 (Figure S11A). As a sensitivity analysis, we recalculated results using mortality risk ratios for post tuberculosis reported by Lee Rodriguez 2020, based on a retrospective cohort study of individuals with and without post-TB, controlling for multiple demographic and clinical risk factors for mortality. To calculate future mortality rates for the cohort under a counter-factual where they had not developed tuberculosis disease in 2019, we divided the mortality rates estimated for the tuberculosis scenario by the *causal* mortality rate ratio for each country.

To account for the reduced quality of life for individuals with post-TB we used disability weights for different COPD severity levels reported by the Global Burden of Disease Study,[3] and use the results of GBD 2015 Chronic Respiratory Disease Collaborators 2017[14] to map from these values to different levels of FEV1% impairment. This produced country-level estimates of the incremental disability weight caused by post-TB that varied between 0.006 and 0.086, with a median of 0.036 (Figure S11B).

Figure S11: Distribution of country-level values for the mortality risk-ratios for RS-TB (Panel A), disability weights for RS-TB (Panel B) mortality risk-ratios for RR-TB (Panel C), and disability weights for RR-TB (Panel D).

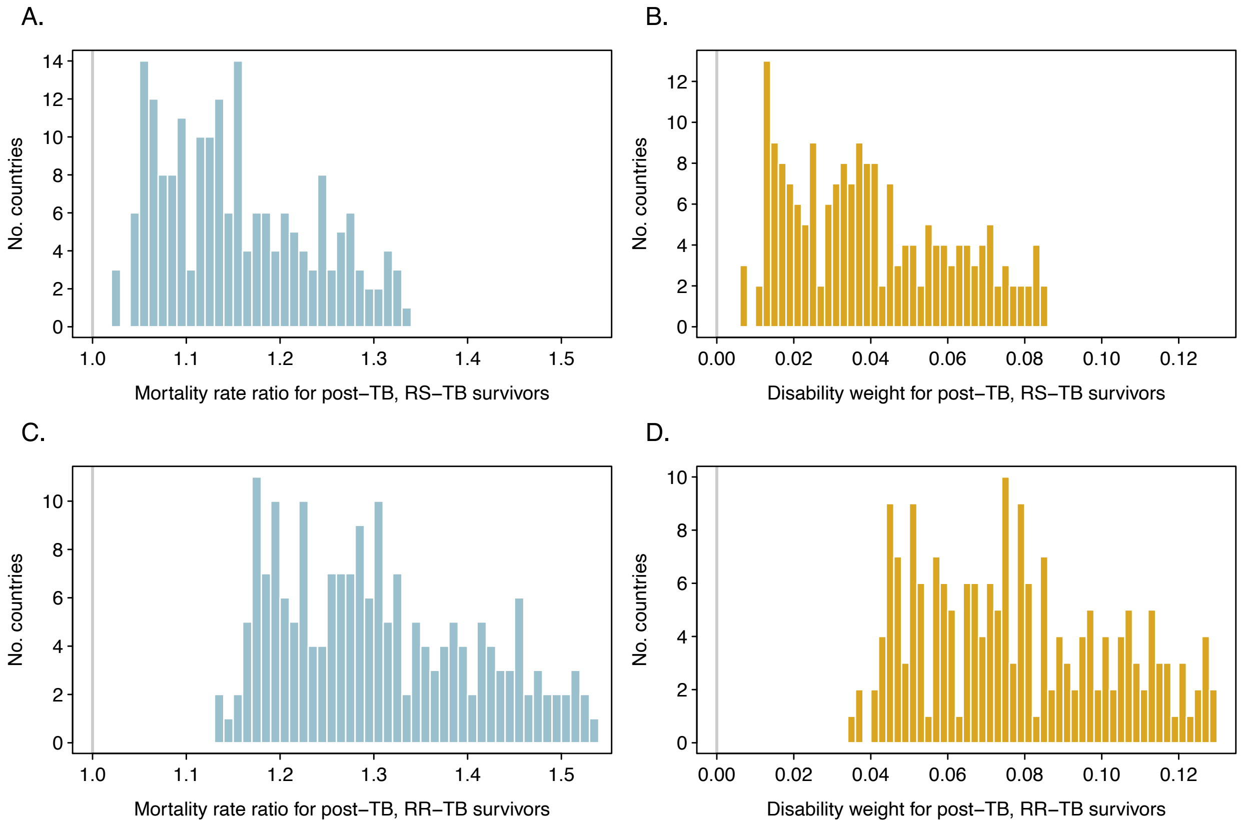

Several studies have described a higher prevalence and severity of tuberculosis sequelae among individuals surviving RR-TB or MDR-TB as compared to RS-TB.[5,15] This additional burden could result from the delayed initiation of an effective treatment regimen, or the need for multiple treatment rounds to achieve cure. As the large majority of tuberculosis cases are RS-TB, we assumed the mortality rate ratios and disability weights described above apply to RS-TB and applied an additional odds ratio of COPD among individuals surviving RR-TB as compared to RS-TB. Given the literature describing the differences between RR-TB and RS-TB survivors is relatively weak, we assumed a uniform distribution for this parameter with a lower bound of 1.0 and an upper bound of 2.73 (mean value = 1.87), based on a systematic review that compared the prevalence of cavitation and other measures of lung function among individuals that had been treated for MDR-TB compared to individuals with non-MDR-TB.[5] Figure S11C shows the resulting distribution of post-TB mortality rate ratios for RR-TB, which range from 1.14 to 1.53, with a median value of 1.29. Figure S11D shows the distribution of disability weights for RR-TB, which range from 0.035 to 0.130, with a median value of 0.075. We also conducted a sensitivity analysis around this value, re-estimating results with the post-TB mortality rate ratios and disability weights for RR-TB assumed to the same as for RS-TB.

Supplement citations

1. WHO Global TB Programme. WHO Global TB Database [http://www.who.int/tb/country/data/download/en/, last accessed Apr 22 2023]. Geveva Switzerland: WHO Global TB Programme; 2023.

2. Evans C, Crawford B. Expert judgement in pharmacoeconomic studies. Guidance and future use. *Pharmacoeconomics* 2000; **17**(6): 545-53.

3. Global Burden of Disease Collaborative Network. Global Burden of Disease Study 2019 (GBD 2019) Disability Weights. (http://ghdx.healthdata.org/record/ihme-data/gbd-2019-disability-weights, accessed Feb 6, 2021). Seattle, USA: Institute for Health Metrics and Evaluation, 2020.

4. Glaziou P AN, Dodd PJ, Dean A, Floyd K. Methods used by WHO to estimate the global burden of TB disease. Geneva Switzerland: Global TB Programme, World Health Organization, 2023.

5. Meghji J, Simpson H, Squire SB, Mortimer K. A Systematic Review of the Prevalence and Pattern of Imaging Defined Post-TB Lung Disease. *PLoS One* 2016; **11**(8): e0161176.

6. Romanowski K, Baumann B, Basham CA, Khan FA, Fox GJ, Johnston JC. Long-term all-cause mortality in people treated for tuberculosis: a systematic review and meta-analysis. *The Lancet Infectious Diseases* 2019; **19**(10): 1129-37.

7. Duong M, Islam S, Rangarajan S, et al. Mortality and cardiovascular and respiratory morbidity in individuals with impaired FEV(1) (PURE): an international, community-based cohort study. *Lancet Glob Health* 2019; **7**(5): e613-e23.

8. Lee-Rodriguez C, Wada PY, Hung Y-Y, Skarbinski J. Association of mortality and years of potential life lost with active tuberculosis in the United States. *JAMA network open* 2020; **3**(9): e2014481-e.

9. Byrne AL, Marais BJ, Mitnick CD, Lecca L, Marks GB. Tuberculosis and chronic respiratory disease: a systematic review. *Int J Infect Dis* 2015; **32**: 138-46.

10. UNAIDS. AIDSinfo Epidemiological Estimates Database [retrieved from https://aidsinfo.unaids.org/, Feb 6 2021]. Geneva, Switzerland: UNAIDS, 2021.

11. World Health Organization. Guidance for the surveillance of drug resistance in tuberculosis. 2021.

12. Brasil Ministério da Saúde. Departamento de Informática do Sistema Único de Saúde. Sistema de Informação de Agravos de Notificação (SINAN) [https://datasus.saude.gov.br/transferencia-de-arquivos/]: Brasilia, Brazil, 2022.

13. Allwood BW, Myer L, Bateman ED. A systematic review of the association between pulmonary tuberculosis and the development of chronic airflow obstruction in adults. *Respiration* 2013; **86**(1): 76-85.

14. GBD 2015 Chronic Respiratory Disease Collaborators. Global, regional, and national deaths, prevalence, disability-adjusted life years, and years lived with disability for chronic obstructive pulmonary disease and asthma, 1990-2015: a systematic analysis for the Global Burden of Disease Study 2015. *Lancet Respir Med* 2017; **5**(9): 691-706.

15. Ivanova O, Hoffmann VS, Lange C, Hoelscher M, Rachow A. Post-tuberculosis lung impairment: systematic review and meta-analysis of spirometry data from 14 621 people. *European Respiratory Review* 2023; **32**(168): 220221.
