# Supplementary material for: Global burden of disease due to rifampicin-resistant tuberculosis: a mathematical modeling analysis
Source: Nat Commun. 2023 Oct 4;14:6182. doi: 10.1038/s41467-023-41937-9 (PMC10550952; doi:10.1038/s41467-023-41937-9)
Supplement: Supplementary file 3 — Reporting Summary [file 41467_2023_41937_MOESM3_ESM.pdf]

nature portfolio

Corresponding author(s): Nicolas A Menzies

Last updated by author(s): Sep 12, 2023

Reporting Summary

Nature Portfolio wishes to improve the reproducibility of the work that we publish. This form provides structure for consistency and transparency in reporting. For further information on Nature Portfolio policies, see our Editorial Policies and the Editorial Policy Checklist.

Statistics

For all statistical analyses, confirm that the following items are present in the figure legend, table legend, main text, or Methods section.

n/a	Confirmed	
☐	☒	The exact sample size (*n*) for each experimental group/condition, given as a discrete number and unit of measurement
☒	☐	A statement on whether measurements were taken from distinct samples or whether the same sample was measured repeatedly
☒	☐	The statistical test(s) used AND whether they are one- or two-sided<br>*Only common tests should be described solely by name; describe more complex techniques in the Methods section.*
☐	☒	A description of all covariates tested
☐	☒	A description of any assumptions or corrections, such as tests of normality and adjustment for multiple comparisons
☐	☒	A full description of the statistical parameters including central tendency (e.g. means) or other basic estimates (e.g. regression coefficient) AND variation (e.g. standard deviation) or associated estimates of uncertainty (e.g. confidence intervals)
☒	☐	For null hypothesis testing, the test statistic (e.g. *F*, *t*, *r*) with confidence intervals, effect sizes, degrees of freedom and *P* value noted<br>*Give P values as exact values whenever suitable.*
☐	☒	For Bayesian analysis, information on the choice of priors and Markov chain Monte Carlo settings
☒	☐	For hierarchical and complex designs, identification of the appropriate level for tests and full reporting of outcomes
☒	☐	Estimates of effect sizes (e.g. Cohen's *d*, Pearson's *r*), indicating how they were calculated

Our web collection on statistics for biologists contains articles on many of the points above.

Software and code

Policy information about availability of computer code

Data collection	No software was used for data collection.
Data analysis	Analyses were conducted in R (version 4.2.3). Analytic code are available at https://zenodo.org/record/8339608.

For manuscripts utilizing custom algorithms or software that are central to the research but not yet described in published literature, software must be made available to editors and reviewers. We strongly encourage code deposition in a community repository (e.g. GitHub). See the Nature Portfolio guidelines for submitting code & software for further information.

Data

Policy information about availability of data

All manuscripts must include a data availability statement. This statement should provide the following information, where applicable:

- Accession codes, unique identifiers, or web links for publicly available datasets
- A description of any restrictions on data availability
- For clinical datasets or third party data, please ensure that the statement adheres to our policy

Most data used in this study are publicly available. UN Population Division life tables are available here: https://population.un.org/wpp/. WHO TB data (country reported data and estimated values) are available here: http://www.who.int/tb/country/data/download/en/. UNAIDS HIV estimates are available here: https://aidsinfo.unaids.org/. Global Burden of Disease Study disability weights are available here: http://ghdx.healthdata.org/record/ihme-data/gbd-2019-disability-weights. Brazil TB data are available here: https://datasus.saude.gov.br/transferencia-de-arquivos/. Copies of these datasets are available at https://doi.org/10.5281/

zenodo.8339608. Historical data from routine drug resistance surveillance and surveys are not publicly available due to privacy issues, and were shared by WHO for this analysis. Summary estimates of these data are published each year in WHO's Global TB Report (https://www.who.int/teams/global-tuberculosis-programme/tb-reports/global-tuberculosis-report-2022). Data can be provided on request to Dr Anna Dean at deanan@who.int, with an expected response time of 2 weeks.

Research involving human participants, their data, or biological material

Policy information about studies with human participants or human data. See also policy information about sex, gender (identity/presentation), and sexual orientation and race, ethnicity and racism.

Reporting on sex and gender	Study results are reported stratified by sex.
Reporting on race, ethnicity, or other socially relevant groupings	Study results were not stratified by race or other socially-constructed variable. Data to stratify results by these characteristics were not available for the analysis.
Population characteristics	Study results are stratified by age, country, TB disease stage, drug resistance status, and HIV status.
Recruitment	Not applicable -- study involved no recruitment.
Ethics oversight	Not applicable -- study represents non Human Subjects Research

Note that full information on the approval of the study protocol must also be provided in the manuscript.

Field-specific reporting

Please select the one below that is the best fit for your research. If you are not sure, read the appropriate sections before making your selection.

☒ Life sciences ☐ Behavioural & social sciences ☐ Ecological, evolutionary & environmental sciences

For a reference copy of the document with all sections, see nature.com/documents/nr-reporting-summary-flat.pdf

Life sciences study design

All studies must disclose on these points even when the disclosure is negative.

Sample size	Study included all country-specific datasets available for analysis.
Data exclusions	We excluded countries with less than 10 estimated TB cases for 2020, as estimates for these countries can be unreliable.
Replication	Not applicable -- study represented a mathematical modelling analysis, so replication of the main analysis would produce identical results. In sensitivity analyses we examine and report the robustness of results to alternative analytic assumptions
Randomization	We randomly sampled from probability distributions for each analytic input to propagate uncertainty through the analysis, using a Monte Carlo simulation approach.
Blinding	Blinding was not applicable to this study as this study did not address an analytic question that involved assignment to treatment arms or other experimental groups where blinding would be possible.

Reporting for specific materials, systems and methods

We require information from authors about some types of materials, experimental systems and methods used in many studies. Here, indicate whether each material, system or method listed is relevant to your study. If you are not sure if a list item applies to your research, read the appropriate section before selecting a response.

Materials & experimental systems

n/a	Involved in the study	
☒	☐	Antibodies
☒	☐	Eukaryotic cell lines
☒	☐	Palaeontology and archaeology
☒	☐	Animals and other organisms
☒	☐	Clinical data
☒	☐	Dual use research of concern
☒	☐	Plants

Methods

n/a	Involved in the study	
☒	☐	ChIP-seq
☒	☐	Flow cytometry
☒	☐	MRI-based neuroimaging
